# Production of Graphite Nanoplatelets via Functionalized Polyketone-Assisted Diels–Alder Chemistry: Evidence of Reduced Layer Thickness and Enhanced Exfoliation Efficiency

**DOI:** 10.3390/polym17101333

**Published:** 2025-05-14

**Authors:** Ricardo Cisternas, Jaime Orellana, Nataly Silva, Jonathan Correa-Puerta, Andrea Pucci, Ranjita K. Bose, Francesco Picchioni, Esteban Araya-Hermosilla, Rodrigo Araya-Hermosilla

**Affiliations:** 1 Programa de Magíster en Química con Mención en Tecnología de los Materiales, Universidad Tecnológica Metropolitana, Santiago 7800003, Chile; ricardo.cisternasr@utem.cl; 2 Programa de Doctorado en Ciencia de los Materiales e Ingeniería de Procesos, Universidad Tecnológica Metropolitana, Santiago 7800003, Chile; jaime.orellanao@utem.cl; 3 Facultad de Diseño, Universidad del Desarrollo, Avenida Plaza 680, Las Condes 7610658, Chile; nrsilva@udd.cl; 4 Departamento de Física, Universidad Técnica Federico Santa María, Av. España 1680, Valparaíso 2390123, Chile; jonathan.correa@usm.cl; 5 Dipartimento di Chimica e Chimica Industriale, Università di Pisa, Via Moruzzi 13, 56124 Pisa, Italy; andrea.pucci@unipi.it; 6 Department of Chemical Product Engineering, Engineering and Technology Institute Groningen (ENTEG), University of Groningen, Nijenborgh 4, 9747AG Groningen, The Netherlands; r.k.bose@rug.nl (R.K.B.); f.picchioni@rug.nl (F.P.); 7 Facultad de Ciencias Físicas y Matemáticas, Departamento de Ingeniería Química, Biotecnología y Materiales, Universidad de Chile, Beauchef 851, Santiago 8370456, Chile; 8 Instituto Universitario de Investigación y Desarrollo Tecnológico (IDT), Universidad Tecnológica Metropolitana, Ignacio Valdivieso 2409, San Joaquín 8940577, Chile

**Keywords:** polyketone, Paal–Knorr reaction, graphite, Diels–Alder reaction, polymer intercalation, exfoliation, graphite nanoplates, nanocomposite

## Abstract

This study introduces an efficient and scalable method for the top-down exfoliation of graphite into graphite nanoplatelets (GNPs) using polyketones (PKs) functionalized with Diels–Alder (DA) active groups. Leveraging the reversible covalent interactions facilitated by furan and thiophene moieties in PK, combined with melt-mixing and shear force, this process achieves significant exfoliation while preserving the structural integrity of the resulting material. Thermal and rheological analyses demonstrate enhanced interfacial adhesion and stability within polymer composites attributed to the DA-driven interactions between functionalized PK and graphite. Comparative evaluations demonstrate that furan-functionalized PK exhibits superior exfoliation efficiency, highlighting its potential for producing high-quality exfoliated graphite suitable for advanced nanocomposite applications that require enhanced thermal, mechanical, and electrical properties. This method seamlessly integrates sustainability with industrial scalability, offering significant advancements in developing GNP-based materials.

## 1. Introduction

Graphene is a single layer of carbon atoms arranged in a honeycomb lattice. It has gained significant attention due to its exceptional properties, including a high surface area of 2418 m^2^/g and remarkable strength, which surpasses those of diamond and steel. Additionally, graphene demonstrates superior electrical conductivity of 1.4 × 10^6^ S/cm and thermal conductivity of 5000 W/m·K. These characteristics make them ideal for various applications, including supercapacitors, antibacterial scaffolds, and transparent electronics [1,2,3,4,5,6,7]. The performance of graphene in various applications is influenced by its manufacturing method, which can be categorized as either bottom-up or top-down. Bottom-up techniques, such as chemical vapor deposition (CVD), involve constructing graphene from smaller molecular components. In contrast, top-down methods, like exfoliation, produce graphene by breaking down bulk graphite [8,9,10,11,12,13].

Graphite, a precursor of graphene, consists of stacked carbon layers connected by van der Waals forces [14,15,16]. Graphite can be exfoliated into graphene through various techniques, including liquid-phase exfoliation (LPE), electrochemical exfoliation, and mechanical forces [17,18,19,20,21,22,23,24,25]. The crystal borders of graphite influence the types and quantities of defects that occur during exfoliation, which can impact the conductivity of graphene [26,27,28]. Additionally, graphite can act as a diene and a dienophile in Diels–Alder (DA) reactions, allowing graphite intercalation with organic molecules and then enhancing the exfoliation process. This reactivity enables the reversible formation of covalent DA bonds, which can be used to optimize exfoliation techniques [29,30,31]. Moreover, polymers such as polypropylene and polystyrene can intercalate graphite, facilitating graphite exfoliation into graphene materials and improving graphene-polymer composites for large-scale production [32,33,34,35,36,37,38,39,40,41].

Recent studies have explored methods for intercalating graphite with DA-functionalized polymers to enhance the compatibility and properties of graphene. For instance, DA-modified graphite has been demonstrated to enhance the thermal conductivity and provide good dispersion within polymer matrices [42,43,44,45,46]. Optimizing the DA-functional group density and processing conditions is essential for enhancing the intercalation and exfoliation efficiency, ensuring the production of high-quality graphene derivatives for industrial applications [47].

In a previous investigation, we achieved a high level of multi-walled carbon nanotube (MWCNT) exfoliation using polyketone (PK) functionalized with aromatic and hydroxyl pendant groups [48,49]. The resulting nanocomposites demonstrated an effective percolative network at low MWCNTs wt.% loading and a highly stable electrical response after several heating and cooling cycles. Similarly, PK was functionalized with diene furan groups, allowing the exfoliation of MWCNTs through reversible covalent interactions via Diels–Alder chemistry [47]. Moreover, PK functionalized with amide and carboxylic groups [50] and furan groups [51] were used to exfoliate RGO using supramolecular and the Diels–Alder reactions, respectively. However, the exfoliation of RGO was not successful. Given the mixed results presented by the authors, this research focuses on a comprehensive investigation of the exfoliation of graphite to generate graphene derivatives by employing the melt-mixing exfoliation method assisted by Diels–Alder functionalized PKs.

PK is a promising starting polymer for the preparation of functional polymers through the Paal–Knorr chemical modification approach. This is due to the highly reactive 1,4-di-carbonyl moieties, which react with primary amines to form N-substituted pyrrole units [52]. The Paal–Knorr reaction is versatile and can be performed in bulk without the need for catalysts, resulting in high yields with water as the only by-product [53]. Additionally, it is tolerant to various primary amine derivatives, making it an efficient method for producing polymers with a wide range of desired pendant functional groups [49,51,52,53]. The resulting functional polymers have a variety of applications, including self-healing materials [54], emulsions with adhesive properties [55], and coating materials [56].

**Figure 1:**
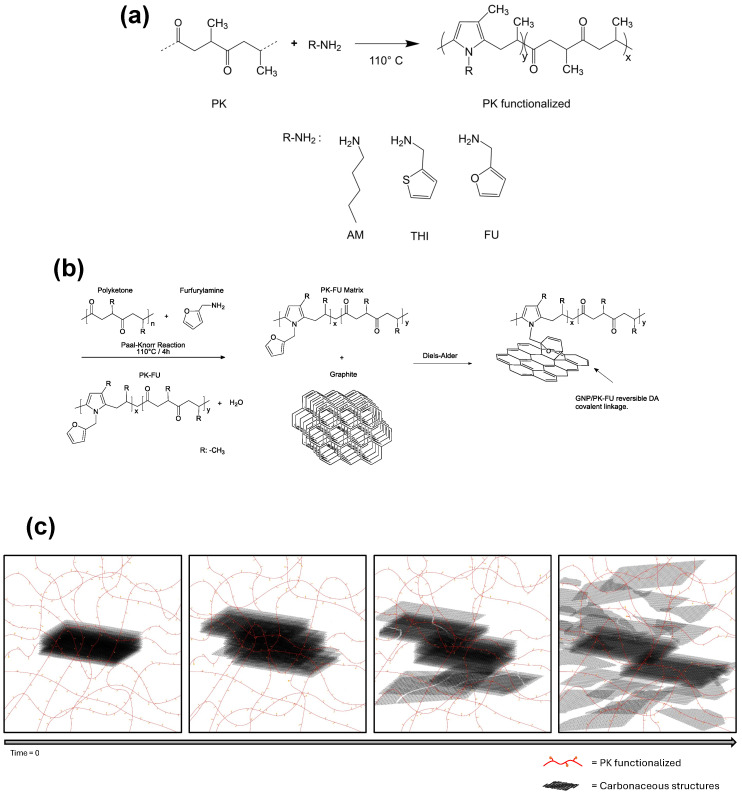
(**a**) Schematic representation of the Paal–Knorr reaction and polyketone products bearing AM (PKAM), THI (PKTHI), and FU (PKFU) functional groups. (**b**) Diels–Alder reaction between PK-FU and graphite, its exfoliation into GNP, and the composite formation showing DA covalent linkages. (**c**) Schematic representation of the progressive exfoliation of graphite into GNP over time by mixing with functionalized PK (red lines PK, orange dots THI or FU), aided by shear force.

This study presents a simple and efficient method for graphite exfoliation through an in-situ melt-mixing process utilizing polyketones (PKs) functionalized with diene active groups. The polymers employed include an aliphatic polyketone synthesized via the copolymerization of propylene and carbon monoxide (CO), alongside PKs modified with various functional groups: aliphatic moieties (PKAM), furan (PKFU), and thiophene (PKTHI) (Figure 1a). In this approach, furan and thiophene serve as reactive diene molecules in the Diels–Alder (DA) reaction with graphite, which acts as a dienophile, facilitating efficient exfoliation [47]. The primary objective of this work is to investigate and compare the exfoliation efficiency of furan and thiophene functional groups in promoting the Diels–Alder reaction with graphite. This study aims to determine which diene achieves superior exfoliation performance, ultimately advancing the development of high-quality exfoliated graphite for diverse applications.

## 2. Experimental Section

### 2.1. Materials

Alternating aliphatic PK made of propylene and carbon monoxide were synthesized according to a reported procedure (Mw 5337 Da) [57]. 1-aminopentane (Am) (Sigma-Aldrich, 99%, Schnelldorf, Germany), 2-Thiophenemethylamine (THI) (Sigma-Aldrich, 96%, Hoeilaart, Belgium), NaCl (Sigma-Aldrich, ≥99.0%, Germany), CHCl_3_ (J.T. Baker, 99.8%, Phillipsburg, NJ, USA), CDCl_3_ (Sigma-Aldrich, 99.8 AtomD, Milwaukee, WI, USA) for ^1^H-NMR analysis, DMF (Supelco, ≥99.8%, Darmstadt, Germany), N-methylpyrrolidone (NMP) (Sigma-Aldrich, 99%, Schnelldorf, Germany), THF (Supelco, ≥99.8%, Darmstadt, Germany), were used as received. Filters of 0.45 μm (PVDF, 47 mm, Durapore, Jaffrey, NH, USA) and 0.22 μm (PTFE, 30 mm, Biolfil, Shantou, China) were used for solvent/solute separation. Graphite Powder (Gr) (20 μm, synthetic, Sigma-Aldrich, Buchs, Switzerland). 2-Aminomethylfuran (FU) (Sigma-Aldrich ≥ 99%, Schnelldorf, Germany) was freshly distilled before use.

### 2.2. Methods

#### 2.2.1. Functionalization of Polyketone with Aliphatic, Furan, and Thiophene Groups

The polyketone (PK) was modified using either 1-aminopentane (referred to as PKAM, the control sample), 2-aminomethylfuran (PKFU), or 2-thiophenemethylamine (PKTHI) through the Paal–Knorr reaction in bulk. The aim was to achieve a total carbonyl conversion of 80% (refer to Figure 1, Figure S1 for the experimental setup, and Table S1 for the stoichiometry of the reactants and calculations). In the standard procedure, the unmodified PK was placed inside a 250 mL Teflon reactor equipped with an overhead mechanical stirrer (Ultra Torque Model BDC1850, Caframo, Georgian Bluffs, ON, Canada) and a thermoregulated oil bath [52]. The reactor was heated up to 110 °C until the polymer reached a liquid state. Subsequently, AM, FU, or THI were added dropwise to the reactor within the first 30 min. The stirring was maintained at a constant speed of 400 rpm using a Teflon impeller (4 Flat Blade Impeller), and the reaction time was set to 4 h after the addition of the final drop of the different amine compounds. Throughout the reaction, the color of the reaction mixture changed progressively from light yellow to dark brown due to the conversion of the carbonyl moieties of the PK backbone into pyrrole groups (Figure 1a).

After the reaction, the polymers were cooled to room temperature. Then, PKAM, PKFU, and PKTHI were dissolved in chloroform and purified by solvent extraction with brine water 0.2 M [49]. The resulting products were then poured into a Teflon Petri dish and placed in a vacuum oven at 50 °C for 48 h to remove any trace of solvent. The polymers were analyzed using ATR–FTIR and ^1^H-NMR spectroscopy to ensure that no solvent was present and to confirm the functionalization of PK with AM, THI, and FU. The samples were stored in amber sample bottles at room temperature for further analysis and use. The carbonyl conversion (Cco), i.e., the molar fraction of 1,4-dicarbonyl units that reacted via the Paal–Knorr reaction, was calculated based on elemental analysis (Formulas (S1)–(S5), Table S1). In addition, TGA, DSC, and rheometric analysis were also performed.

#### 2.2.2. Graphite Exfoliation Assisted by Polyketones

Graphite exfoliation was accomplished by mixing molten unmodified PK and functionalized PK (PKAM, PKFU, or PKTHI) at 5 wt.% of graphite. Consequently, the samples were named PKGr, PKAMGr, PKFUGr, and PKTHIGr. The mixing reaction was performed in a Teflon reactor (130 mL) immersed in an oil bath thermoregulated by a hot plate with a T-1000 thermal sensor (Fulda, Germany). The reactor was equipped with an overhead mechanical mixer (Ultra Torque Model BDC1850, Caframo) with a three-blade paddle-type flow impeller of stainless steel (Figure S2).

In the general procedure, the functionalized polymers PKAM, PKFU, PKTHI, or pristine PK were placed inside a reactor that had been previously heated to 70 °C. Once the polymer was softened (≈30 min), 5 wt.% of graphite was added to start the mixing at 400 rpm for 18 h to exfoliate graphite into graphite nanoplates (Figure 1c). A temperature of 70 °C was chosen to favor the DA reaction between the diene functional groups (FU or THI) and graphite as a dienophile; 18 h corresponds to the time needed to crosslink the composite during mixing (Figure S3). Heating the crosslinked systems to 150 °C allowed for de-crosslinking (i.e., melting) and composite liquefaction through the r-DA reaction.

The crosslinked composites were dissolved in DMF at a ratio of 1:10 (composite: DMF) in a round-bottom flask that was placed in an oil bath. The setup was equipped with a reflux cooling system and a magnetic stirrer. The mixture was then heated to 150 °C and stirred at 400 rpm for 1 h.

This specific condition was selected to facilitate the separation of the components using a high temperature. Next, the flask was rapidly cooled in liquid nitrogen (N_2_(l)) to quench the Diels–Alder reaction. The resulting solution, containing both the polymer and exfoliated graphite, was filtered at room temperature. This filtration process involved two stages: initially using a PVDF membrane with a 0.45 μm pore size, followed by a PTFE membrane with a 0.22 μm pore size. This process allowed the separating of the graphitic structures from the polymer solution. For further analysis, the filters containing the graphitic structures were immersed in NMP to resuspend the samples and achieve a good dispersion of the exfoliated graphite [58]. Around 0.5 mg/mL (samples from 0.45 μm filters) and 0.1 mg/mL (samples from 0.22 μm filters) were used for dynamic light scattering (DLS) and scanning transmission electron microscope (STEM) analysis. After removing NMP in a vacuum oven at 60 °C for 48 h, dried exfoliated graphite samples were used for Raman spectroscopy, scanning electron microscopy (SEM), and atomic force microscopy (AFM) analysis.

#### 2.2.3. Mass Yield of Exfoliated Graphite

The PKFUGr system was chosen and subjected to centrifugation at 13 K rpm instead of filtration to measure the mass yield of the exfoliated graphite. This approach leverages the principle that exfoliated graphite settles at the bottom of the centrifuge tube, while the low-molecular-weight modified polyketone remains suspended in the supernatant.

This method facilitated the effective separation of the components, which were subsequently dried and weighed. The purity of the separated materials was verified by thermogravimetric analysis (TGA) (Figure S15), and the effective exfoliation was verified by scanning electron microscopy (SEM) (Figure S16).

### 2.3. Characterization

The functionalized polymers’ elemental composition was determined using an Elemental Vario Micro Cube (Milano, Italy) for nitrogen, carbon, and hydrogen. The ATR–FTIR spectra of the polymers were recorded using a Nicolet iS5 (Fitchburg, WI, USA) within the 4000–500 cm^−1^ range and averaged over 32 scans. The ^1^H-NMR spectra of the polymers were recorded at room temperature in CDCl_3_ solution with a Bruker Avance III HD 400 MHz (Bruker, Billerica, MA, USA), using the residual solvent peak as an internal reference. The thermal degradation of the polymers was analyzed via thermogravimetric analysis (TGA) using a Mettler Toledo TGA/DSC 1 (Chemnitz, Germany) under nitrogen flux from 30 °C to 550 °C at 10 °C/min. DSC analysis was performed under an N_2_ atmosphere. The samples (3 to 7 mg) were weighed in an aluminum pan and then sealed. The samples were first heated from −30 to 160 °C, followed by cooling to −30 °C. Four cycles were performed, with each heating and cooling step incorporating an isotherm of 10 min, and the heating and cooling rates were set to 10 °C/min throughout the DSC measurements. Rheology measurements were performed using a SmartPave 102e Anton Paar (Greencity, Graz, Austria) instrument. The samples were analyzed in the melt state using a parallel plate system with a geometry PP50 and 1 mm gap. Temperature ramps were performed in oscillation mode with 0.2% strain and 1 Hz, from 110 to 50 °C at a rate of 3.5 °C/min. Owing to the high fluidity of the polyketone, temperature ramps were also carried out between 80 and 10 °C. Raman spectroscopy was performed using a confocal InVia Raman microscope (Renishaw, Wotton-under-Edge, England). The wavelength of the excitation laser was 532 nm. Sample preparation involved depositing a droplet of the exfoliated graphite material in NMP solvent onto silicon mounted on a standard pin.

The morphology of the exfoliated graphite material was analyzed using a Gemini 360 ZEISS field-emission scanning electron microscope (FE-SEM) (Oberkochen, Germany). The instrument is equipped with an FE-Gun and an “Inlens” detector and operates at a 5 keV accelerating voltage with variable magnification. Statistical analyses of the particle size were performed at a magnification of 1.5 K for graphite particles resuspended from 0.45 µm filters and at a magnification of 20 K for graphite particles resuspended from 0.22 µm filters. The sample preparation was the same as that used for Raman spectroscopy.

The size of the exfoliated graphite was evaluated using dynamic light scattering (DLS, Zetasizer Nano ZS90 (Worcestershire, UK)) at 25 °C. A laser beam operating at 633 nm was used, and detection was performed at an angle of 90°. Before each analysis, the samples were left in a sonication bath for 60 s and then settled for 60 s before starting the measurement. NMP was used as the solvent. Each measurement was carried out in triplicate. Data collection and analysis were performed using the Zetasizer software 8.02 (Malvern Panalytical, Worcestershire, UK). The mass concentration of exfoliated graphite and the volume of solvent used for each sample were 0.5 mg/mL for the 0.45 μm filters and 0.1 mg/mL for the 0.22 μm filters.

The exfoliated graphite material’s internal structure and layer density were analyzed using an INSPECT F50 FEI instrument (FEI, Hillsboro, OR, USA) equipped with a STEM detector. The examination was conducted at an accelerating voltage of 10 keV, with variable magnification settings.

Sample preparation involved depositing a droplet of the resuspended material from the 0.22 filter onto a formvar/carbon 300 mesh copper grid. To examine the size and morphology of the exfoliated graphite material, a CoreAFM atomic force microscope (AFM) (Liestal, Switzerland) was used, employing Tap300AI-G probes (BudgetSensors^®^, Sofia, Bulgaria) with variable scales. Statistical analysis was performed on the obtained images. The preparation consisted of dissolving the PKFUGr sample (obtained from 0.45 and 0.22 µm filters) in THF due to its relatively rapid evaporation at a ratio of 1:3 (sample:THF). The mixture was then subjected to r-DA reaction conditions at 150 °C, with a stirring speed of 400 rpm for 10 min. After the reaction period, the sample was cooled in N_2_(l). Once the sample reached room temperature, 1 mL of the sample was uniformly sprayed onto a silicon wafer for subsequent analysis. X-ray diffraction analysis was performed to study the graphite composite using a Bruker D8 Advance (Karlsruhe, Germany), Ka1 Cu. All structural parameters were obtained from the literature [59,60]. The d-spacings were calculated using Bragg’s equation:

(1) $$d_{002}=\frac{\lambda }{2\sin\theta_{002}}$$

where *λ* is the radiation wavelength (0.15406 nm) and *θ* is the diffraction angle.

## 3. Results and Discussion

### 3.1. Functionalization of Polyketone with Aliphatic, Furan, and Thiophene Groups

**Figure 2:**
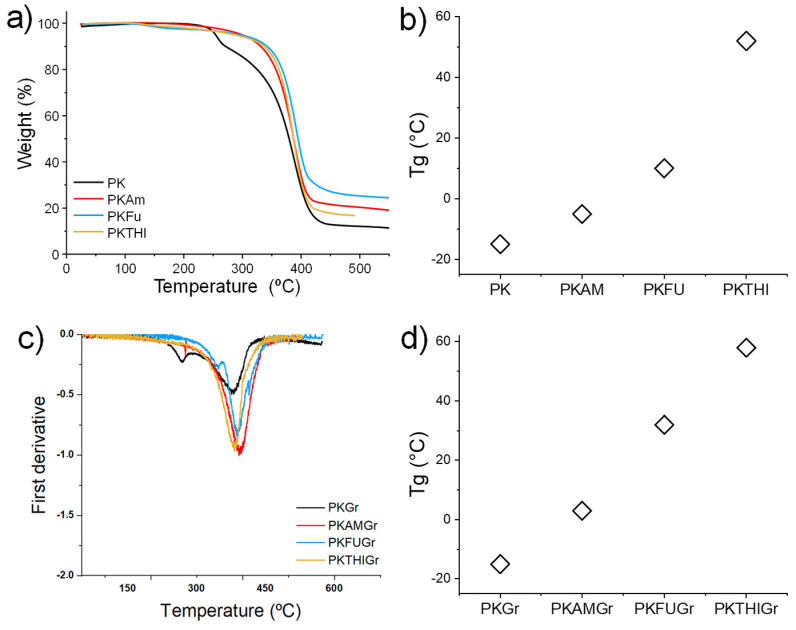
(**a**) Thermogravimetric curves and (**b**) T_g_ values as determined by DSC analysis of pristine PK, functionalized PK, PKAM, PKFU, and PKTHI. (**c**) First derivative of the thermogravimetric curve of the PKs/graphite (5%) composite thermograms and (**d**) T_g_ values as determined by DSC analysis.

We successfully functionalized polyketone (PK) with 1-aminopentane (PKAM), 2-thiophenamine (PKTHI), and 2-aminomethylfuran (PKFU) through chemical modification using the Paal–Knorr reaction (Figure 2). PKAM was used as the control polymer in this study.

The functionalization of the PK resulted in carbonyl conversions (Cco%) of 38, 60, and 46 for PKAM, PKFU, and PKTHI, respectively, as estimated from elemental analysis (see Table S2). ATR-FT-IR (Figure S4) and ^1^H-NMR (Figure S5) spectroscopy confirmed the functional grafting of the groups onto the PK backbone.

We studied the thermal stability of the functionalized polymers by conducting thermogravimetric analysis (TGA) on samples ranging from room temperature to 550 °C. Figure 2a displays the decomposition temperatures of the polymers. The unmodified polyketone (PK) exhibited a two-step decomposition process, with an initial decomposition temperature of 225 °C and a second step at 388 °C. In contrast, the functionalized PK did not display the initial decomposition step at 225 °C, as observed for the unmodified PK. Instead, it showed a single decomposition step near the second decomposition temperature of the unmodified PK. This behavior indicates the enhanced thermal stability of PK following functionalization.

The increase in PK thermal stability is likely due to the decreased number of polyketone carbonyl groups after the formation of the pyrrole ring via the Paal–Knorr reaction. PKAM, PKFU, and PKTHI decomposed at 389 °C, 392 °C, and 387 °C, respectively [40]. This can be explained by the higher stability of the heterocyclic compounds and the formation of carbon-rich residues instead of volatile compounds. This trend aligns with the results shown in Table S2, where PKFU demonstrates the highest carbonyl conversion, followed by PKTHI, and finally PKAM [61,62].

These data are highly relevant to our research since the exfoliation experiments’ temperature conditions fall within the range in which the polymers do not experience thermal decomposition. DSC analysis (Figure S6) was also performed on the synthesized polymers to investigate their thermal transitions, particularly the glass transition temperatures of these non-crystalline materials (Figure 2b). The functionalization of the polymers increased their glass transition temperature (T_g_) compared to that of the unmodified PK, which exhibited a T_g_ of −15 °C. The increase in the polymer’s T_g_ is primarily due to the enhanced rigidity of the polymer’s main chains caused by the presence of pyrrole groups. The T_g_ values for the functionalized polymers PKAM, PKFU, and PKTHI were observed at −5 °C, 10 °C, and 52 °C, respectively. These T_g_ values underscore the significant structural changes resulting from the grafting of furan and thiophene groups in PKFU and PKTHI, respectively.

Notably, PKTHI exhibited a higher T_g_ value than PKFU, despite achieving a lower carbonyl conversion to pyrroles during the Paal–Knorr reaction. This behavior is primarily attributed to the higher electronegativity of the sulfur atoms in the thiophene (THI) pendant groups of PKTHI [63].

We conducted rheological analyses on pristine and functionalized polyketones (PKs) to evaluate the effects of different functional groups on the processability and mechanical properties of the materials (Figure S7). Measurements were conducted above 50 °C, as the pristine PK sample exhibited slippage between the parallel plates of the rheometer at lower temperatures, which affected the data accuracy. Consequently, the storage (G′) and loss (G″) moduli of the polymers were compared at 60 °C (Table 1). The results indicate a clear increase in the rigidity of PK upon functionalization with amine (AM), furan (FU), and thiophene (THI) groups. Introducing pyrrole groups through amine functionalization stiffens the polymer matrix due to the increased intermolecular interactions and restricted segmental motion imparted by the pyrrole units. This effect is consistent with the findings in the literature, where pyrrole groups in the polymer backbone enhance rigidity by increasing the network density and limiting chain flexibility [49]. Adding pendant furan groups (PKFU) further enhances rigidity compared to PKAM. This enhancement may be attributed to the furan rings’ inherent rigidity and the steric hindrance they introduce, which limits chain mobility and contributes to a higher modulus. A similar trend is observed in polymers containing pyrrole heterocycles in the main chain and pendant thiophene groups (THI), where the larger atomic volume of the heterocycle leads to higher storage and loss moduli. Generally, we observe typical thermoplastic behavior, with both the storage and loss moduli decreasing as the temperature increases [64].

### 3.2. Polymer/Graphite Composites

Graphite exfoliation was achieved by first softening pristine or functionalized polyketone (PK, PKAM, PKFU, or PKTHI) at 70 °C, followed by the addition of graphite. A temperature of 70 °C was chosen to ensure the softening of the polymer, which helps to intercalate the polymer chains between the graphite interlayers. In addition, polymers with diene moieties may covalently interact with graphite layers or edges via the Diels–Alder (DA) reaction [32]. Under the selected experimental conditions, the polymer/graphite mixture underwent crosslinking after 18 h, resulting in the formation of composites, as shown in Figure S3. This behavior suggests the occurrence of covalent interactions between functionalized PKFU and graphite, consistent with previous findings on PKFU–MWCNT composites reported by the same authors [47].

### 3.3. Thermal Characterization

The composites were then analyzed using DSC (Figure S8). The functionalized polymer composites showed a general trend of increasing T_g_ compared to their respective polymers (Figure 2c), indicating the occurrence of effective interactions between the polymer and graphite. The composites PKAMGr, PKFUGr, and PKTHIGr exhibited glass transition temperatures (T_g_) of 3 °C, 32 °C, and 58 °C, respectively, reflecting increases of 8 °C, 22 °C, and 6 °C compared to their corresponding unmodified polymers. The notable T_g_ enhancement in PKFUGr and PKTHIGr can be attributed to specific interactions between the pendant diene groups (furan and thiophene) and the graphitic material, which likely promote stronger interfacial adhesion and restrict the polymer chain mobility. Similarly, the T_g_ increase observed in PKAMGr suggests a potential interaction between the graphitic filler and the pyrrole ring in the polymer structure, further indicating the compatibility and interaction between the components in the composite matrix [65,66]. In contrast, the control sample PKGR exhibited no change in T_g_ (−15 °C), suggesting the absence of an interaction between the polymer matrix and graphite components.

The thermal degradation behavior of the composites was systematically analyzed, with the PKGr sample serving as a benchmark. PKGr exhibited a thermal profile comparable to that of pristine PK, with an initial decomposition temperature of 268 °C and a secondary decomposition phase at 380 °C. These results suggest minimal interaction between the polymer matrix and the graphite material in the absence of functionalization.

In contrast, the functionalized polymer composites containing 5 wt.% of graphite (PKAMGr, PKFUGr, PKTHIGr) exhibited consistent decomposition temperatures of 390 °C, 388 °C, and 382 °C, respectively. This uniformity indicates that the incorporation of functionalized graphite does not adversely affect the thermal stability of the polymer, even after the graphite exfoliation process. The stability of the decomposition temperatures aligns with the expected behavior of Diels–Alder bonds, which are thermally reversible (retro-Diels–Alder, r-DA) and break down at high temperatures, leading to polymer decomposition while leaving the graphite material intact. Moreover, the addition of graphite appears to increase the decomposition temperature of the polymers compared to that of pristine PK. This enhancement suggests that the graphitic filler acts as a radical scavenger, inhibiting the early stages of polymer degradation [67,68]. These findings are particularly significant as they confirm that functionalized graphite contributes to improved thermal stability without compromising the structural integrity of the polymer under high-temperature conditions, which is a critical requirement for its intended applications.

### 3.4. Rheology Characterization

Rheological analyses were conducted on the PK composites (Figure S9), revealing notable increases in the moduli upon adding graphitic material (Table 2). However, this increase in the control sample (PKGr) was minimal compared to that of the functionalized polymer composites, indicating limited interaction between the unmodified PK and graphite.

Interestingly, the enhanced modulus observed in PKAMGr compared to that of PKAM suggests a degree of interaction with the graphitic filler, likely mediated by the pyrrole ring in the polymer structure [66]. The most pronounced improvements were observed in the PKFUGr and PKTHIGr composites, consistent with the higher density of diene groups in these functionalized polymers. These diene groups facilitate stronger interfacial interactions with the graphitic material, significantly enhancing the mechanical properties of the composite. The increase in the moduli of PKFUGr and PKTHIGr was so substantial that the materials exhibited solid-like behavior at lower temperatures, rendering conventional rheological analysis less suitable. This behavior underscores the effectiveness of functionalization in promoting strong polymer-graphite interactions, leading to the formation of reinforced composites with superior mechanical performance.

### 3.5. Microscopy Characterization

**Figure 3:**
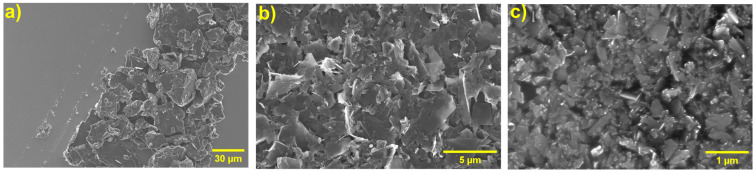
SEM images obtained at a magnification of (**a**) Gr (500×) filter 0.45 µm, (**b**) PKFUGr (1500×) filter 0.45 µm, and (**c**) PKFUGr (20 K×) Filter 0.22 µm.

The composite materials were further characterized using SEM, Raman spectroscopy, XRD, STEM (Figures S16–S20), and AFM to investigate their structural and morphological properties. SEM analysis provided insights into the morphology and particle size of graphite before and after the exfoliation process. Figure 3 illustrates the graphite material retained on 0.45 µm filters before and after exfoliation (refer to the Experimental Section for details). Pristine graphite (Figure 3a) displayed large spherical particles with an average area of approximately 450 µm^2^. A similar morphology and size were observed in graphite exfoliated with unmodified PK (Figure S19a), indicating minimal interaction between the pristine polymer and the graphite. In contrast, graphite treated with functionalized polymers (PKAM, PKFU, and PKTHI) exhibited significant changes in particle size and morphology, confirming effective exfoliation. The SEM images (Figure 3b and Figure S19b,c) reveal that the exfoliated graphite particles adopted a flake-like morphology and displayed significantly reduced average particle areas. Further analysis of the graphite retained on 0.22 µm filters showed nanometric-sized graphite structures (Figure 3c and Figure S20a,b), indicating that the exfoliation process successfully produced graphite nanoplatelets. These findings demonstrate that functionalized polymers play a critical role in facilitating the exfoliation of graphite, leading to significant reductions in particle size and altered morphology.

The histograms presented in Figures S10–S14, generated from the analysis of the SEM micrographs of various samples, illustrate the distribution of the exfoliated graphite material across the analyzed areas.

The graphite materials exfoliated with the functionalized polymers show a significant reduction in size compared to PKGr (490 µm^2^) and Gr (450 µm^2^). Specifically, the graphite exfoliated with PKAM predominantly presents flake areas in the range of 10 µm^2^, thus suggesting that the Gr exfoliation occurs via intercalation and chemical interactions between the pyrrole group and Gr. Similarly, graphite exfoliated with PKFU and PKTHI displayed flake areas with distributions centered at 3.83 µm^2^ and 3.17 µm^2^, respectively. This reduction in particle size can be attributed to the higher density of diene groups present in these functionalized polymers. These diene groups play a critical role as intercalation and exfoliation agents, facilitating the penetration of polymer chains between graphite layers. The slightly smaller flake area observed in the PKTHI-exfoliated graphite may be due to the higher electronegativity of the sulfur atoms in the thiophene groups, which could enhance the interaction with graphite, further aiding the exfoliation process.

### 3.6. AFM Characterization

**Figure 4:**
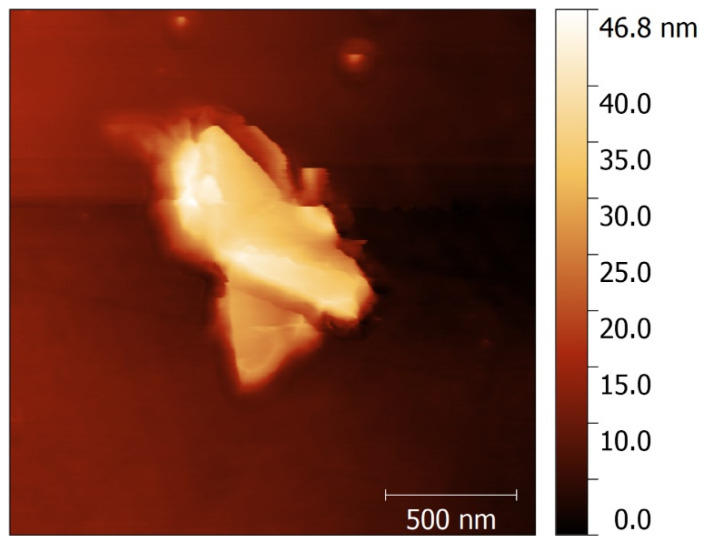
The AFM image of 2 µm × 2 µm deposited on silicon wafer PK0FUGr 0.45 µm filter where exfoliated graphitic structures with random sizes are observed.

Atomic Force Microscopy (AFM) analysis was performed on the most effective exfoliation system, PKFUGr, to estimate the number of layers in the exfoliated graphite material. The material was filtered, dried, and resuspended in tetrahydrofuran (THF). Histograms derived from the AFM images indicated that the graphite material retained on the 0.45 µm filter exhibited a high degree of size polydispersity. The largest graphite fragments were beyond the measurement range of the AFM, leading to an analysis focusing on smaller fragments within this sample. In comparison, the smaller fragments on the 0.45 µm filter (Figure 4) were longer than those retained on the 0.22 µm filter. Across both samples (0.45 and 0.22 µm), the exfoliated graphite material exhibited approximately 80–120 stacked exfoliated graphite layers. Notably, the material filtered at 0.22 µm demonstrated a smaller size polydispersity, suggesting a more uniform distribution of fragment sizes in this sample [25,69]. Comparing these findings with existing scientific literature, we classify these structures as exfoliated graphite nanoplatelets (xGNPs). This classification is supported by the morphology and properties observed, which are consistent with graphite nanoplates described in other studies [69,70,71].

### 3.7. Raman Spectroscopy

We analyzed the exfoliated graphite material using Raman spectroscopy. Raman spectroscopy provides robust signals from graphene derivatives. The main characteristic bands can be observed at 1330 cm^−1^ (D-band) associated with edges and defects (sp^3^ carbons), 1580 cm^−1^ (G-band) assigned to sp^2^ carbons, and the bands in the range of 2400–2750 cm^−1^ G+ and 2D band, where the 2D band is the second-order overtone of the D-band. In addition, the intensity ratio between the D and G bands (I_D_/I_G_) is crucial for indicating defect levels in graphite derivatives [72,73].

**Figure 5:**
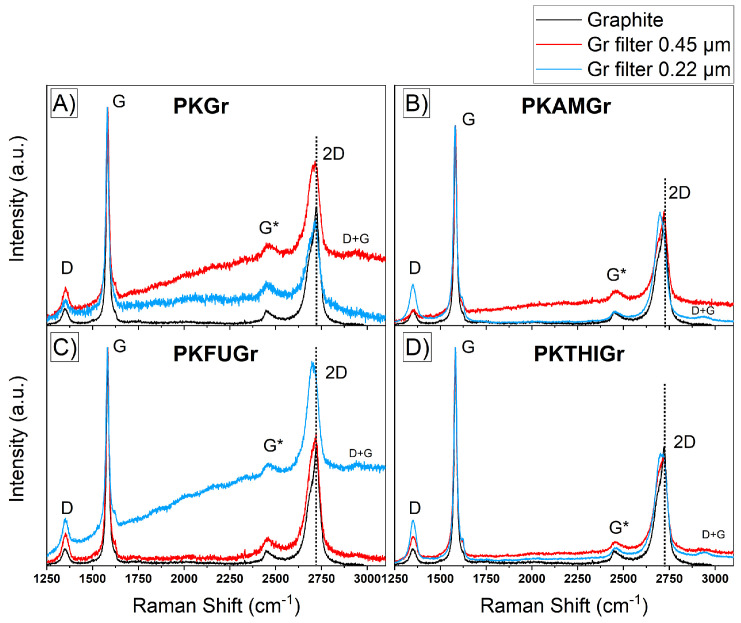
Raman spectra of Gr (Black), Exfoliated graphite retained in 0.45 µm filter (Red), Exfoliated graphite retained in 0.22 µm filter (Blue). (**A**) PKGR, (**B**) PKAMGr, (**C**) PKFUGr, and (**D**) PKTHIGr.

Figure 5 presents the Raman spectra of the graphite materials obtained after the exfoliation process. The samples were categorized into two groups based on the filtration membranes used: 0.45 µm and 0.22 µm. Notably, the samples filtered through the 0.45 µm membrane exhibited an increased I_D_/I_G_ ratio compared to that of the original graphite. This ratio, which reflects the intensity of the D-band (associated with defects and disorder in the graphitic structure) relative to the G-band (indicative of in-plane vibrations of sp^2^-bonded carbon atoms), suggests a higher concentration of structural defects in the material. Table 3 summarizes these ratios. The increased defect density observed in the 0.45 µm membrane group likely arose from the mechanical and chemical processes involved in exfoliation, which introduced imperfections into graphite layers. These defects could be attributed to the breaking of graphitic bonds or the intercalation of polymer functional groups, both of which disrupt the pristine structure of graphite. The results highlight the effectiveness of the exfoliation process in generating graphite nanoplatelets (GNPs) with altered structural characteristics, which potentially enhances their reactivity and compatibility with composite materials.

The observed increase in the I_D_/I_G_ band ratio for the PKAMGr sample is particularly noteworthy and possibly suggests the formation of Diels–Alder (DA) cycloadducts between the pyrrole rings present in the main chain of the polymer and the graphitic material, which likely occurred in other graphitic materials such as MWCNTs [74]. This chemical interaction introduces structural defects at the graphitic surface, which are detected as an increase in D-band intensity relative to the G-band. In contrast, the increase in the I_D_/I_G_ ratio for the control sample (PKGr) appears to have resulted from mechanical defects introduced during the exfoliation process rather than chemical interactions. These defects arise from the physical disruption of graphite layers without significant bonding or interaction with the polymer matrix. The I_2D_/I_G_ ratio, on the other hand, provides insight into the stacking order and layer structure of the graphitic material. An I_2D_/I_G_ ratio of approximately 2 is characteristic of monolayer graphene, indicating a high degree of structural uniformity. Ratios less than 2 suggest the presence of stacked graphene layers, with increasing stacking resulting in progressively lower I_2D_/I_G_ ratios. This ratio is a critical metric for assessing the quality and exfoliation efficiency of the graphitic material, as it distinguishes between few-layer graphene and bulk-like stacked structures [75,76]. Similarly, for samples resuspended in the 0.22 µm filter, the I_D_/I_G_ ratio exhibited the same behavior as the samples from the 0.45 µm filter. However, in the PKFUGr system, the I_2D_/I_G_ ratio increased to 0.9 compared to 0.6 for the original graphite powder. This increase suggests a decrease in the number of stacked, exfoliated graphite layers. Another notable finding was the change observed in the 2D signal for all exfoliated graphite. A redshift in the 2D and G* positions of the Raman bands and a broadening of these bands were observed (dashed lines). These changes indicate a decrease in the number of layers of exfoliated graphite sheets [76]. This result reinforces the hypothesis of an exfoliating interaction between the PKAM and the graphitic material. In contrast, the control sample PKGr shows a 2D signal with no changes, confirming the absence of exfoliation or reduction in the graphitic stacked material.

### 3.8. X-Ray Diffraction (XRD)

**Figure 6:**
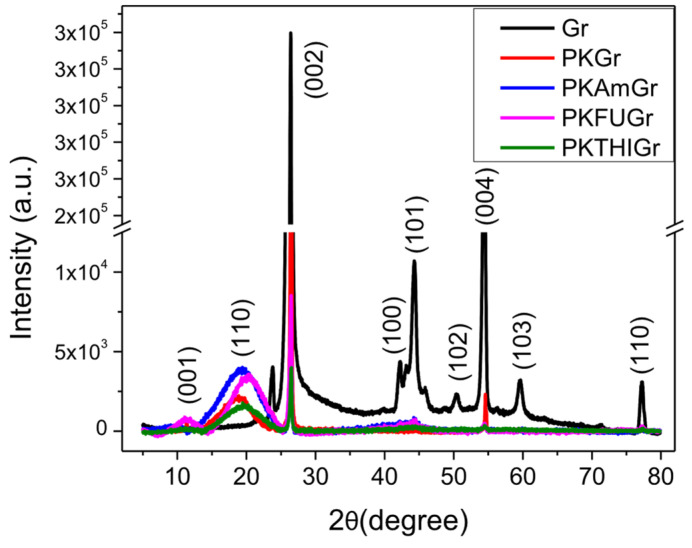
X-ray powder diffraction patterns of Gr, composites: PKGr, PKAMGr, PKFUGr, PKTHIGr between 5 < 2θ < 80°.

X-ray diffraction (XRD) analysis is a useful technique for characterizing the exfoliation of graphite into graphene derivatives. This technique provides information on layer spacing and interlayer distances, indicating exfoliation. The exfoliation indicates a reduction in the Gr crystallinity, as evidenced by the weakening or disappearance of the sharp (002) peak characteristic of graphite. The latter helps to assess the degree of exfoliation of Gr. Additionally, XRD can reveal whether the exfoliated layers retain their stacking order or become randomly oriented. Figure 6 shows the XRD patterns of graphite and its composites with PK and functionalized PK (AM, FU, and THI). The figure displays the broad PK reflection at 20° and the graphite diffractogram, highlighting the characteristic peaks and their corresponding plane assignments.

For pristine graphite, a strong and sharp peak is observed at 2θ = 26.4°, corresponding to the (002) plane. This peak, originating from polyaromatic layers, indicates a highly ordered graphitic structure with a basal spacing of d_002_ = 0.337 nm (Table 4) [77], which further confirms the low defect density through the low I_D_/I_G_ ratio, consistent with the Raman spectroscopy results. The next prominent peak at 2θ = 54.4° corresponds to the (004) plane, reflecting the stacking of higher order polyaromatic layers. Another significant peak, centered at 44.3°, is associated with the (101) plane, while the peak at 42.4°, corresponding to the (100) plane, helps define the longitudinal dimensions of the structural elements of graphite [59]. Additionally, low-intensity peaks are observed at 50.5°, 59.6°, and 77.4°, with planes equivalent to (102), (103), and (110), respectively [78].

The XRD spectra of graphitic materials obtained after exfoliation processes reveal a noticeable decrease in the intensity of the (002) plane as compared to that of the pristine graphite [79,80]. The reduction in the intensity of the θ_002_ plane is attributed to the significant disruption of the graphitic structure caused by exfoliation and the restacking or reorganization of exfoliated layers during the composite preparation process. This phenomenon reflects the complex interplay between the exfoliated layers and the PK matrix, which introduces new interlayer interactions with the polyketone matrix [81] (PDF card #00-02-0212) [82].

Due to the structural and compositional differences introduced during the exfoliation and PK integration processes, exfoliation comparisons will be focused exclusively on PK-modified samples to ensure a consistent and meaningful analysis. Interestingly, in the functionalized samples (PKAMGr, PKFUGr, and PKTHIGr), the intensity of the (002) peak decreases, which reflects the enhanced exfoliation achieved through the functionalization of PK. Incorporating functional groups such as Am, FU, and THI introduces additional interactions at the edges and surfaces of the graphitic layers, effectively disrupting interlayer interactions and reducing the stacking order [83,84]. Raman spectra reveal a higher I_D_/I_G_ ratio in these samples, confirming the increased defect density introduced by the functionalization. At the same time, variations in I_2D_/I_G_ suggest differences in the degree of stacking among the functionalized samples.

Finally, a comparative table assessing various graphite exfoliation methods is essential to evaluate the potential of our technology for the low-cost, scalable production of exfoliated graphite (see comparative data in Table S4). Compared to existing exfoliation methods, our PKFU-assisted polymer exfoliation strategy demonstrates a unique balance between high yield, structural integrity, process scalability, and environmental compatibility. With a mass yield of 87.9%, our approach significantly outperforms conventional techniques, such as liquid-phase exfoliation (20–30%) and mechanical milling (40–60%), and even matches or surpasses electrochemical exfoliation methods in terms of efficiency. While other methods may achieve thinner layers, they often require intensive processing steps, hazardous solvents, or costly purification, making them less viable for large-scale applications. In contrast, our method employs a melt-mixing process with minimal solvent use and no need for aggressive sonication or electrodes, enhancing both the economic and environmental profiles of the technique. Although the resulting exfoliated graphite exhibits a slightly higher thickness (~30–40 nm), the preserved lateral dimensions and structural quality are well suited for nanocomposite integration. Importantly, the Diels–Alder functionalization strategy enables strong interfacial compatibility with polymers, which is critical for composite performance, an aspect that is often overlooked in purely physical exfoliation methods. This positions our method as a scalable and sustainable alternative for producing exfoliated graphite tailored for advanced materials engineering, especially in applications where interfacial properties and mechanical reinforcement are as important as the layer count itself.

## 4. Conclusions

This work demonstrates a sustainable and scalable approach for exfoliating graphite into graphite nanoplatelets (GNPs) using polyketones functionalized with Diels–Alder-active groups. Among the tested systems, furan-functionalized polyketones (PKFU) exhibited superior exfoliation efficiency, achieving smaller and more uniform exfoliated graphite particles with enhanced structural properties, as validated by Raman spectroscopy and other analytical techniques. The reversible Diels–Alder reaction plays a pivotal role in facilitating exfoliation by enabling strong interfacial adhesion between the functionalized polyketones and graphite, while also suggesting potential recyclability, thereby underscoring the versatility and efficiency of this approach. The integration of pyrrole groups into the polymer backbone further enhanced intercalation and exfoliation, ensuring the structural integrity of graphite under mild thermal conditions (50–150 °C). The application of shear forces during melt-mixing enabled the effective exfoliation and uniform distribution of exfoliated graphite within polymer matrices, resulting in composites with improved thermal, mechanical, and electrical properties. This sustainable and scalable process offers significant potential for industrial applications in aerospace, electronics, energy storage, and advanced nanocomposite materials. By minimizing defects and enhancing the compatibility of exfoliated graphite with polymer matrices, this method provides a pathway for the large-scale production of high-performance exfoliated graphite-based composites, while addressing environmental and economic considerations.

## Figures and Tables

**Table 1:** Functionalized polymer moduli at 60 °C.

Samples	Storage Modulus (MPa)	Loss Modulus (MPa)
PK	8.04 × 10^−8^	2.33 × 10^−5^
PKAM	5.30 × 10^−6^	3.02 × 10^−4^
PKFU	1.64 × 10^−5^	5.21 × 10^−4^
PKTHI	4.81 × 10^−5^	2.16 × 10^−3^

**Table 2:** Composite moduli at 60 °C.

Samples	Storage Modulus (MPa)	Loss Modulus (MPa)
PKGr	1.45 × 10^−6^	5.44 × 10^−5^
PKAMGr	7.31 × 10^−4^	6.71 × 10^−4^
PKFUGr	1.06 × 10^−3^	1.48 × 10^−2^
PKTHIGr	2.39 × 10^−1^	4.49 × 10^−1^

**Table 3:** I_D_/I_G_ and I_2D_/I_G_ band ratios.

Samples	Filter 0.45 μm		Filter 0.22 μm	
	I_D_/I_G_	I_2D_/I_G_	I_D_/I_G_	I_2D_/I_G_
Gr	0.12	0.6	-	-
PKGr	0.38	0.7	0.32	0.6
PKAMGr	0.21	0.6	0.25	0.6
PKFUGr	0.21	0.6	0.43	0.9
PKTHIGr	0.21	0.5	0.25	0.5

**Table 4:** Structural parameters of graphite samples obtained from XRD patterns.

Sample	Graphite			PK
	2θ_002_ (°)	Intensity	*d*_002_ (nm)	2θ_110_ (°)
Gr	26.4	290,957	0.337	-
PKGr	26.5	14,336	0.336	18.9
PKAMGr	26.5	5998	0.336	19.7
PKFUGr	26.5	8527	0.336	20.2
PKTHIGr	26.5	3999	0.336	19.6

## Data Availability

The original contributions presented in this study are included in the article/Supplementary Material. Further inquiries can be directed to the corresponding author.

## Supplementary Materials

The following supporting information can be downloaded at: https://www.mdpi.com/article/10.3390/polym17101333/s1. References [85,86,87,88,89,90,91] are cited in the supplementary materials.

## References

[1] Miculescu M., Thakur V.K., Miculescu F., Voicu S.I. (2016). Graphene-Based Polymer Nanocomposite Membranes: A Review. Polym. Adv. Technol..

[2] Shahryari Z., Yeganeh M., Gheisari K., Ramezanzadeh B. (2021). A Brief Review of the Graphene Oxide-Based Polymer Nanocomposite Coatings: Preparation, Characterization, and Properties. J. Coat. Technol. Res..

[3] Shah R., Kausar A., Muhammad B., Shah S. (2015). Progression from Graphene and Graphene Oxide to High Performance Polymer-Based Nanocomposite: A Review. Polym. Plast. Technol. Eng..

[4] Meyer J.C., Geim A.K., Katsnelson M.I., Novoselov K.S., Booth T.J., Roth S. (2007). The Structure of Suspended Graphene Sheets. Nature.

[5] Pal N., Dubey P., Gopinath P., Pal K. (2017). Combined Effect of Cellulose Nanocrystal and Reduced Graphene Oxide into Poly-Lactic Acid Matrix Nanocomposite as a Scaffold and Its Anti-Bacterial Activity. Int. J. Biol. Macromol..

[6] Kymakis E., Savva K., Stylianakis M.M., Fotakis C., Stratakis E. (2013). Flexible Organic Photovoltaic Cells with In Situ Nonthermal Photoreduction of Spin-Coated Graphene Oxide Electrodes. Adv. Funct. Mater..

[7] Lu C.H., Yang H.H., Zhu C.L., Chen X., Chen G.N. (2009). A Graphene Platform for Sensing Biomolecules. Angew. Chem. Int. Ed. Engl..

[8] Gutiérrez-Cruz A., Ruiz-Hernández A.R., Vega-Clemente J.F., Luna-Gazcón D.G., Campos-Delgado J. (2022). A Review of Top-down and Bottom-up Synthesis Methods for the Production of Graphene, Graphene Oxide and Reduced Graphene Oxide. J. Mater. Sci..

[9] Bonaccorso F., Lombardo A., Hasan T., Sun Z., Colombo L., Ferrari A.C. (2012). Production and Processing of Graphene and 2d Crystals. Mater. Today.

[10] Sun L., Yuan G., Gao L., Yang J., Chhowalla M., Gharahcheshmeh M.H., Gleason K.K., Choi Y.S., Hong B.H., Liu Z. (2021). Chemical Vapour Deposition. Nat. Rev. Methods Primers.

[11] Yan Y., Nashath F.Z., Chen S., Manickam S., Lim S.S., Zhao H., Lester E., Wu T., Pang C.H. (2020). Synthesis of Graphene: Potential Carbon Precursors and Approaches. Nanotechnol. Rev..

[12] Rudrapati R., Rudrapati R. (2020). Graphene: Fabrication Methods, Properties, and Applications in Modern Industries. Graphene Production and Application.

[13] Sumdani M.G., Islam M.R., Yahaya A.N.A., Safie S.I. (2021). Recent Advances of the Graphite Exfoliation Processes and Structural Modification of Graphene: A Review. J. Nanopart. Res..

[14] Mukhopadhyay P., Gupta R.K. (2013). Graphite, Graphene, and Their Polymer Nanocomposites.

[15] Zhao L., Tang J., Zhou M., Shen K. (2022). A Review of the Coefficient of Thermal Expansion and Thermal Conductivity of Graphite. New Carbon Mater..

[16] Backes C., Abdelkader A.M., Alonso C., Andrieux-Ledier A., Arenal R., Azpeitia J., Balakrishnan N., Banszerus L., Barjon J., Bartali R. (2020). Production and Processing of Graphene and Related Materials. 2D Mater..

[17] Kairi M.I., Dayou S., Kairi N.I., Bakar S.A., Vigolo B., Mohamed A.R. (2018). Toward High Production of Graphene Flakes—A Review on Recent Developments in Their Synthesis Methods and Scalability. J. Mater. Chem. A Mater..

[18] Guo Y., Peng F., Wang H., Huang F., Meng F., Hui D., Zhou Z. (2018). Intercalation Polymerization Approach for Preparing Graphene/Polymer Composites. Polymers.

[19] Shi Z., He P., Wang N., Liu Y., Chen X., Li Y., Ding G., Yu Q., Xie X. (2022). Bubble-Mediated Mass Production of Graphene: A Review. Adv. Funct. Mater..

[20] Olabi A.G., Abdelkareem M.A., Wilberforce T., Sayed E.T. (2021). Application of Graphene in Energy Storage Device—A Review. Renew. Sustain. Energy Rev..

[21] Aghamohammadi H., Eslami-Farsani R., Torabian M., Amousa N. (2020). Recent Advances in One-Pot Functionalization of Graphene Using Electrochemical Exfoliation of Graphite: A Review Study. Synth. Met..

[22] Toyoda M., Hou S., Huang Z.H., Inagaki M. (2023). Exfoliated Graphite: Room Temperature Exfoliation and Their Applications. Carbon Lett..

[23] Liu F., Wang C., Sui X., Riaz M.A., Xu M., Wei L., Chen Y. (2019). Synthesis of Graphene Materials by Electrochemical Exfoliation: Recent Progress and Future Potential. Carbon Energy.

[24] Rana N., Narang J., Chauhan A. (2024). Advancing Frontiers: Graphene-Based Nano-Biosensor Platforms for Cutting-Edge Research and Future Innovations. Indian J. Microbiol..

[25] Bianco A., Cheng H.M., Enoki T., Gogotsi Y., Hurt R.H., Koratkar N., Kyotani T., Monthioux M., Park C.R., Tascon J.M.D. (2013). All in the Graphene Family—A Recommended Nomenclature for Two-Dimensional Carbon Materials. Carbon.

[26] Dhar S., Barman A.R., Ni G.X., Wang X., Xu X.F., Zheng Y., Tripathy S., Ariando, Rusydi A., Loh K.P. (2011). A New Route to Graphene Layers by Selective Laser Ablation. AIP Adv..

[27] Pirzado A.A., Le Normand F., Romero T., Paszkiewicz S., Papaefthimiou V., Ihiawakrim D., Janowska I. (2019). Few-Layer Graphene from Mechanical Exfoliation of Graphite-Based Materials: Structure-Dependent Characteristics. ChemEngineering.

[28] Komeily-Nia Z., Qu L.T., Li J.L. (2021). Progress in the Understanding and Applications of the Intrinsic Reactivity of Graphene-Based Materials. Small Sci..

[29] Seo J.M., Jeon I.Y., Baek J.B. (2013). Mechanochemically Driven Solid-State Diels–Alder Reaction of Graphite into Graphene Nanoplatelets. Chem. Sci..

[30] Cai M., Thorpe D., Adamson D.H., Schniepp H.C. (2012). Methods of Graphite Exfoliation. J. Mater. Chem..

[31] Schmidt C., Rosen M.E., Caplan D.F., Pines A., Quinton M.F. (1995). Orientation and Motion of Tetrahydrofuran in Graphite Intercalation Compounds. Proton NMR Studies of Cs(THF)1.3C24 and K(THF)2.5C24. J. Phys. Chem..

[32] Torkaman N.F., Kley M., Bremser W., Wilhelm R. (2022). Reversible Functionalization and Exfoliation of Graphite by a Diels–Alder Reaction with Furfuryl Amine. RSC Adv..

[33] Wang Z., Tong J., Li W., Zhang H., Hu M., Chen H., He H. (2021). Highly Enhancing Electrical, Thermal, and Mechanical Properties of Polypropylene/Graphite Intercalation Compound Composites by In Situ Expansion during Melt Mixing. Polymers.

[34] Li Y., Weng S., Niu R., Zhen W., Xu F., Zhu W., Zhang C. (2022). Poly(Vinyl Alcohol)-Assisted Exfoliation of van Der Waals Materials. ACS Omega.

[35] Chen G., Wu C., Weng W., Wu D., Yan W. (2003). Preparation of Polystyrene/Graphite Nanosheet Composite. Polymer.

[36] Bourlinos A.B., Georgakilas V., Zboril R., Steriotis T.A., Stubos A.K., Trapalis C. (2009). Aqueous-Phase Exfoliation of Graphite in the Presence of Polyvinylpyrrolidone for the Production of Water-Soluble Graphenes. Solid. State Commun..

[37] Ho Q.B., Osazuwa O., Modler R., Daymond M., Gallerneault M.T., Kontopoulou M. (2019). Exfoliation of Graphite and Expanded Graphite by Melt Compounding to Prepare Reinforced, Thermally and Electrically Conducting Polyamide Composites. Compos. Sci. Technol..

[38] Nasir A., Kausar A., Younus A. (2015). Polymer/Graphite Nanocomposites: Physical Features, Fabrication and Current Relevance. Polym. Plast. Technol. Eng..

[39] Zotti A., Zuppolini S., Borriello A., Zarrelli M. (2022). Polymer Nanocomposites Based on Graphite Nanoplatelets and Amphiphilic Graphene Platelets. Compos. B Eng..

[40] Kim H., Macosko C.W. (2008). Morphology and Properties of Polyester/Exfoliated Graphite Nanocomposites. Macromolecules.

[41] Díez-Pascual A.M. (2021). Development of Graphene-Based Polymeric Nanocomposites: A Brief Overview. Polymers.

[42] Chen W., Wu K., Liu Q., Lu M. (2020). Functionalization of Graphite via Diels-Alder Reaction to Fabricate Poly (Vinyl Alcohol) Composite with Enhanced Thermal Conductivity. Polymer.

[43] Seo J.M., Baek J.B. (2014). A Solvent-Free Diels–Alder Reaction of Graphite into Functionalized Graphene Nanosheets. Chem. Commun..

[44] Zabihi O., Ahmadi M., Abdollahi T., Nikafshar S., Naebe M. (2017). Collision-Induced Activation: Towards Industrially Scalable Approach to Graphite Nanoplatelets Functionalization for Superior Polymer Nanocomposites. Sci. Rep..

[45] Sarkar S., Bekyarova E., Niyogi S., Haddon R.C. (2011). Diels-Alder Chemistry of Graphite and Graphene: Graphene as Diene and Dienophile. J. Am. Chem. Soc..

[46] Feng Z., Zuo H., Hu J., Yu B., Ning N., Tian M., Zhang L. (2019). In Situ Exfoliation of Graphite into Graphene Nanosheets in Elastomer Composites Based on Diels-Alder Reaction during Melt Blending. Ind. Eng. Chem. Res..

[47] Araya-Hermosilla R., Pucci A., Raffa P., Santosa D., Pescarmona P.P., Gengler R.Y.N., Rudolf P., Moreno-Villoslada I., Picchioni F. (2018). Electrically-Responsive Reversible Polyketone/MWCNT Network through Diels-Alder Chemistry. Polymers.

[48] Migliore N., Polgar L.M., Araya-Hermosilla R., Picchioni F., Raffa P., Pucci A. (2018). Effect of the Polyketone Aromatic Pendent Groups on the Electrical Conductivity of the Derived MWCNTs-Based Nanocomposites. Polymers.

[49] Araya-Hermosilla R., Pucci A., Araya-Hermosilla E., Pescarmona P.P., Raffa P., Polgar L.M., Moreno-Villoslada I., Flores M., Fortunato G., Broekhuis A.A. (2016). An Easy Synthetic Way to Exfoliate and Stabilize MWCNTs in a Thermoplastic Pyrrole-Containing Matrix Assisted by Hydrogen Bonds. RSC Adv..

[50] Araya-Hermosilla E.A., Carlotti M., Picchioni F., Mattoli V., Pucci A. (2020). Electrically-Conductive Polyketone Nanocomposites Based on Reduced Graphene Oxide. Polymers.

[51] Araya-Hermosilla E., Giannetti A., Lima G.M.R., Orozco F., Picchioni F., Mattoli V., Bose R.K., Pucci A. (2021). Thermally Switchable Electrically Conductive Thermoset RGO/PK Self-Healing Composites. Polymers.

[52] Zhang Y., Broekhuis A.A., Stuart M.C.A., Picchioni F. (2008). Polymeric Amines by Chemical Modifications of Alternating Aliphatic Polyketones. J. Appl. Polym. Sci..

[53] Araya-Hermosilla E., Roscam Abbing M., Catalán-Toledo J., Oyarzun-Ampuero F., Pucci A., Raffa P., Picchioni F., Moreno-Villoslada I. (2019). Synthesis of Tuneable Amphiphilic-Modified Polyketone Polymers, Their Complexes with 5,10,15,20-Tetrakis-(4-Sulfonatophenyl)Porphyrin, and Their Role in the Photooxidation of 1,3,5-Triphenylformazan Confined in Polymeric Nanoparticles. Polymer.

[54] Lima G.M.R., Orozco F., Picchioni F., Moreno-Villoslada I., Pucci A., Bose R.K., Araya-Hermosilla R. (2019). Electrically Self-Healing Thermoset MWCNTs Composites Based on Diels-Alder and Hydrogen Bonds. Polymers.

[55] Zhang Y., Broekhuis A.A., Picchioni F. (2007). Aqueous Polymer Emulsions by Chemical Modifications of Thermosetting Alternating Polyketones. J. Appl. Polym. Sci..

[56] Toncelli C., Schoonhoven M.J., Broekhuis A.A., Picchioni F. (2016). Paal-Knorr Kinetics in Waterborne Polyketone-Based Formulations as Modulating Cross-Linking Tool in Electrodeposition Coatings. Mater. Des..

[57] Mul W.P., Dirkzwager H., Broekhuis A.A., Heeres H.J., Van der Linden A.J., Guy Orpen A. (2002). Highly Active, Recyclable Catalyst for the Manufacture of Viscous, Low Molecular Weight, CO–Ethene–Propene-Based Polyketone, Base Component for a New Class of Resins. Inorg. Chim. Acta.

[58] Hernandez Y., Nicolosi V., Lotya M., Blighe F.M., Sun Z., De S., McGovern I.T., Holland B., Byrne M., Gun’ko Y.K. (2008). High-Yield Production of Graphene by Liquid-Phase Exfoliation of Graphite. Nat. Nanotechnol..

[59] Popova A.N. (2017). Crystallographic Analysis of Graphite by X-Ray Diffraction. Coke Chem..

[60] Barnakov C.N., Khokhlova G.P., Malysheva V.Y., Popova A.N., Ismagilov Z.R. (2015). X-Ray Diffraction Analysis of the Crystal Structures of Different Graphites. Solid. Fuel Chem..

[61] Bendrea A.D., Cianga L., Göen Colak D., Constantinescu D., Cianga I. (2023). Thiophene End-Functionalized Oligo-(D,L-Lactide) as a New Electroactive Macromonomer for the “Hairy-Rod” Type Conjugated Polymers Synthesis. Polymers.

[62] Pachariyangkun A., Suda M., Hadsadee S., Jungsuttiwong S., Nalaoh P., Pattanasattayavong P., Sudyoadsuk T., Yamamoto H.M., Promarak V. (2020). Effect of Thiophene/Furan Substitution on Organic Field Effect Transistor Properties of Arylthiadiazole Based Organic Semiconductors. J. Mater. Chem. C Mater..

[63] Calvo-Martín G., Plano D., Sanmartín C. (2022). New Experimental Conditions for Diels–Alder and Friedel-Crafts Alquilation Reactions with Thiophene: A New Selenocyanate with Potent Activity against Cancer. Molecules.

[64] Wang X., Yan Y., Liu T., Su X., Qian L., Song Y., Xu H. (2010). Synthesis and Nonlinear Optical Properties of Polyacetylenes Containing Oxadiazole and Thiophene Pendant Groups with High Thermal Stability. J. Polym. Sci. A Polym. Chem..

[65] Galimberti M., Barbera V., Guerra S., Conzatti L., Castiglioni C., Brambilla L., Serafini A. (2015). Biobased Janus Molecule for the Facile Preparation of Water Solutions of Few Layer Graphene Sheets. RSC Adv..

[66] Barbera V., Brambilla L., Milani A., Palazzolo A., Castiglioni C., Vitale A., Bongiovanni R., Galimberti M. (2018). Domino Reaction for the Sustainable Functionalization of Few-Layer Graphene. Nanomaterials.

[67] Barra G., Guadagno L., Raimondo M., Santonicola M.G., Toto E., Vecchio Ciprioti S. (2023). A Comprehensive Review on the Thermal Stability Assessment of Polymers and Composites for Aeronautics and Space Applications. Polymers.

[68] Tayouri M.I., Estaji S., Mousavi S.R., Salkhi Khasraghi S., Jahanmardi R., Nouranian S., Arjmand M., Khonakdar H.A. (2022). Degradation of Polymer Nanocomposites Filled with Graphene Oxide and Reduced Graphene Oxide Nanoparticles: A Review of Current Status. Polym. Degrad. Stab..

[69] Guerra V., Wan C., Degirmenci V., Sloan J., Presvytis D., Watson M., McNally T. (2019). Characterisation of Graphite Nanoplatelets (GNP) Prepared at Scale by High-Pressure Homogenisation. J. Mater. Chem. C Mater..

[70] Shtein M., Pri-Bar I., Varenik M., Regev O. (2015). Characterization of Graphene-Nanoplatelets Structure via Thermogravimetry. Anal. Chem..

[71] Hope J.T., Sun W., Kewalramani S., Saha S., Lakhe P., Shah S.A., Mason M.J., Green M.J., Hule R.A. (2020). Scalable Production of Graphene Nanoplatelets for Energy Storage. ACS Appl. Nano Mater..

[72] Wu J.B., Lin M.L., Cong X., Liu H.N., Tan P.H. (2018). Raman Spectroscopy of Graphene-Based Materials and Its Applications in Related Devices. Chem. Soc. Rev..

[73] Cançado L.G., Jorio A., Ferreira E.H.M., Stavale F., Achete C.A., Capaz R.B., Moutinho M.V.O., Lombardo A., Kulmala T.S., Ferrari A.C. (2011). Quantifying Defects in Graphene via Raman Spectroscopy at Different Excitation Energies. Nano Lett..

[74] Zydziak N., Yameen B., Barner-Kowollik C. (2013). Diels–Alder Reactions for Carbon Material Synthesis and Surface Functionalization. Polym. Chem..

[75] Malard L.M., Pimenta M.A., Dresselhaus G., Dresselhaus M.S. (2009). Raman Spectroscopy in Graphene. Phys. Rep..

[76] Ferrari A.C., Basko D.M. (2013). Raman Spectroscopy as a Versatile Tool for Studying the Properties of Graphene. Nat. Nanotechnol..

[77] Li K., Liu Q., Cheng H., Hu M., Zhang S. (2021). Classification and Carbon Structural Transformation from Anthracite to Natural Coaly Graphite by XRD, Raman Spectroscopy, and HRTEM. Spectrochim. Acta A Mol. Biomol. Spectrosc..

[78] Li Z.Q., Lu C.J., Xia Z.P., Zhou Y., Luo Z. (2007). X-Ray Diffraction Patterns of Graphite and Turbostratic Carbon. Carbon.

[79] Hadi A., Zahirifar J., Karimi-Sabet J., Dastbaz A. (2018). Graphene Nanosheets Preparation Using Magnetic Nanoparticle Assisted Liquid Phase Exfoliation of Graphite: The Coupled Effect of Ultrasound and Wedging Nanoparticles. Ultrason. Sonochem..

[80] Wang W., Wang Y., Gao Y., Zhao Y. (2014). Control of Number of Graphene Layers Using Ultrasound in Supercritical CO_2_ and Their Application in Lithium-Ion Batteries. J. Supercrit. Fluids.

[81] Orellana J., Araya-Hermosilla E., Pucci A., Araya-Hermosilla R. (2024). Polymer-Assisted Graphite Exfoliation: Advancing Nanostructure Preparation and Multifunctional Composites. Polymers.

[82] Sajid H.M., Afzal H., Irfan M., Saleem M., Jan R., Javed S., Akram M.A. (2022). Design of Multilayered 2D Nanomaterial Composite Structures for EMI Shielding Analysis. ACS Omega.

[83] Cho S., Lee J.S., Jang H., Park S., An J.H., Jang J. (2021). Comparative Studies on Crystallinity, Thermal and Mechanical Properties of Polyketone Grown on Plasma Treated CVD Graphene. Polymers.

[84] Sariyev B., Abdikadyr A., Baitikenov T., Anuarbekov Y., Golman B., Spitas C. (2023). Thermal Properties and Mechanical Behavior of Hot Pressed PEEK/Graphite Thin Film Laminate Composites. Sci. Rep..

[85] Araya-Hermosilla E., Parlanti P., Gemmi M., Mattoli V., Di Pietro S., Iacopini D., Granchi C., Turchi B., Fratini F., Di Bussolo V. (2022). Functionalized Aliphatic Polyketones with Germicide Activity. RSC Adv..

[86] Orozco F., Kaveh M., Santosa D.S., Lima G.M.R., Gomes D.R., Pei Y., Araya-Hermosilla R., Moreno-Villoslada I., Picchioni F., Bose R.K. (2021). Electroactive Self-Healing Shape Memory Polymer Composites Based on Diels–Alder Chemistry. ACS Appl. Polym. Mater..

[87] Pretsch E., Bühlmann P., Badertscher M. (2009). Structure Determination of Organic Compounds: Tables of Spectral Data.

[88] Araya-Hermosilla R., Fortunato G., Pucci A., Raffa P., Polgar L., Broekhuis A.A., Pourhossein P., Lima G.M.R., Beljaars M., Picchioni F. (2016). Thermally Reversible Rubber-Toughened Thermoset Networks via Diels-Alder Chemistry. Eur. Polym. J..

[89] Paton K.R., Varrla E., Backes C., Smith R.J., Khan U., O’Neill A., Boland C., Lotya M., Istrate O.M., King P. (2014). Scalable Production of Large Quantities of Defect-Free Few-Layer Graphene by Shear Exfoliation in Liquids. Nat. Mater..

[90] Achee T.C., Sun W., Hope J.T., Quitzau S.G., Sweeney C.B., Shah S.A., Habib T., Green M.J. (2018). High-Yield Scalable Graphene Nanosheet Production from Compressed Graphite Using Electrochemical Exfoliation. Sci. Rep..

[91] León V., Rodriguez A.M., Prieto P., Prato M., Vázquez E. (2014). Exfoliation of Graphite with Trriazine Derivatives under Ball-Milling Conditions: Preparation of Few-Layer Graphene via Selective Noncovalent Interactions. ACS Nano.

