# Supplementary material for: Production of Graphite Nanoplatelets via Functionalized Polyketone-Assisted Diels–Alder Chemistry: Evidence of Reduced Layer Thickness and Enhanced Exfoliation Efficiency
Source: Polymers (Basel). 2025 May 14;17(10):1333. doi: 10.3390/polym17101333 (PMC12114719; doi:10.3390/polym17101333)
Supplement: Supplementary file 1 [file polymers-17-01333-s001.zip › polymers-3565360-supplementary.pdf]

Supporting information

Production of Graphite Nanoplatelets via Functionalized Polyketone-Assisted Diels–Alder Chemistry: Evidence of Reduced Layer Thickness and Enhanced Exfoliation Efficiency

Ricardo Cisternas[1], Jaime Orellana[2], Nataly Silva[3], Jonathan Correa-Puerta[4], Andrea Pucci[5], Ranjita K. Bose[6], Francesco Picchioni[6], Esteban Araya-Hermosilla [7*], and Rodrigo Araya-Hermosilla [8*]

1 Programa de Magíster en Química con Mención en Tecnología de los Materiales, Universidad Tecnológica Metropolitana, Santiago 7800003, Chile; ricardo.cisternasr@utem.cl
2 Programa de Doctorado en Ciencia de los Materiales e Ingeniería de Procesos, Universidad Tecnológica Metropolitana, Santiago 7800003, Chile; jaime.orellanao@utem.cl
3 Facultad de Diseño, Universidad del Desarrollo, Avenida Plaza 680, Las Condes 7610658, Chile; nrsilva@udd.cl
4 Departamento de Física, Universidad Técnica Federico Santa María, Av. España 1680, Valparaíso 2390123, Chile; jonathan.correa@usm.cl
5 Dipartimento di Chimica e Chimica Industriale, Università di Pisa, Via Moruzzi 13, 56124 Pisa, Italy; andrea.pucci@unipi.it
6 Department of Chemical Product Engineering, Engineering and Technology Institute Groningen (ENTEG), University of Groningen, Nijenborgh 4, 9747AG Groningen, The Netherlands; r.k.bose@rug.nl (R.K.B.); f.picchioni@rug.nl (F.P.)
7 Facultad de Ciencias Físicas y Matemáticas, Departamento de Ingeniería Química, Biotecnología y Materiales, Universidad de Chile, Beauchef 851, Santiago 8370456, Chile
8 Instituto Universitario de Investigación y Desarrollo Tecnológico (IDT), Universidad Tecnológica Metropolitana, Ignacio Valdivieso 2409, San Joaquín 8940577, Chile
* Correspondence: earayahermosilla@ing.uchile.cl (E.A.-H.); rodrigo.araya@utem.cl (R.A.-H.)

S1 Paal-Knorr reaction system setup, stoichiometry of reactants and calculations.

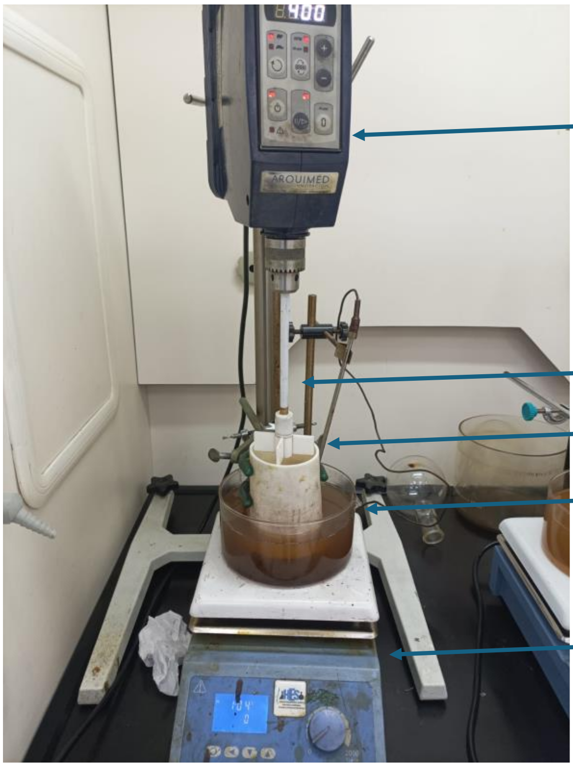

Figure S1. Teflon reactor setup for polyketone chemical modification.

Table S1. Stoichiometric amounts of reactants used for polyketone modification via Paal-Knorr reaction.

Sample	PK (g)	*Ratio NH_2/C=O	Di-carbonyl groups (mmol)	Primary amine group (mmol)	THI (g)	FU (g)	AM (g)	Total (g)
PKAm	15	0.8	85.7	85.7	-	-	7.47	22.47
PKFU	50	0.8	285.7	285.7	-	27.7	-	77.75
PKTHI	50	0.8	285.7	285.7	32.34	-	-	82.34

*carbonyl conversion (Cco).

The carbonyl conversion (Cco) can be calculated by:

$$C_{CO} = \frac{y}{y+x} * 100\% \quad \text{(S1)}$$

where x and y represent the moles of di-ketone and pyrrolic units after conversion, respectively, in a definite mass of product (g_{prod}). y can be calculated as follows:

$$y = \frac{wt_{(N)}}{Am_{(N)}} \quad \text{(S2)}$$

where wt(N) represents the weight in grams of nitrogen in g_{prod}according to elemental analysis and Am(N) is the atomic mass of nitrogen. x can be calculated as follows:

$$x = \frac{g_{prod} - y * M_w^y}{M_w^{pk}} \tag{S3}$$

where M_w^y represents the molecular weight of the pyrrolic functionalized unit and M_w^{pk} the molecular weight of the di-ketone unit (140 g/mol). The conversion efficiency η can be defined as the ratio between the carbonyl conversion C_{CO} according to the moles of dicarbonyl in the feed C_{CO}^{feed}:

$$\eta = \frac{C_{CO}}{C_{CO}^{feed}} * 100 \tag{S4}$$

The $C_{CO}^{feed}$is calculated as follows:

$$C_{CO}^{feed} = \frac{Mol_{amine}}{Mol_{PK0}} * 100 \tag{S5}$$

with Mol_{amine} representing the moles of amine compounds and Mol_{PK0} the moles of di-carbonyl units in the feed.

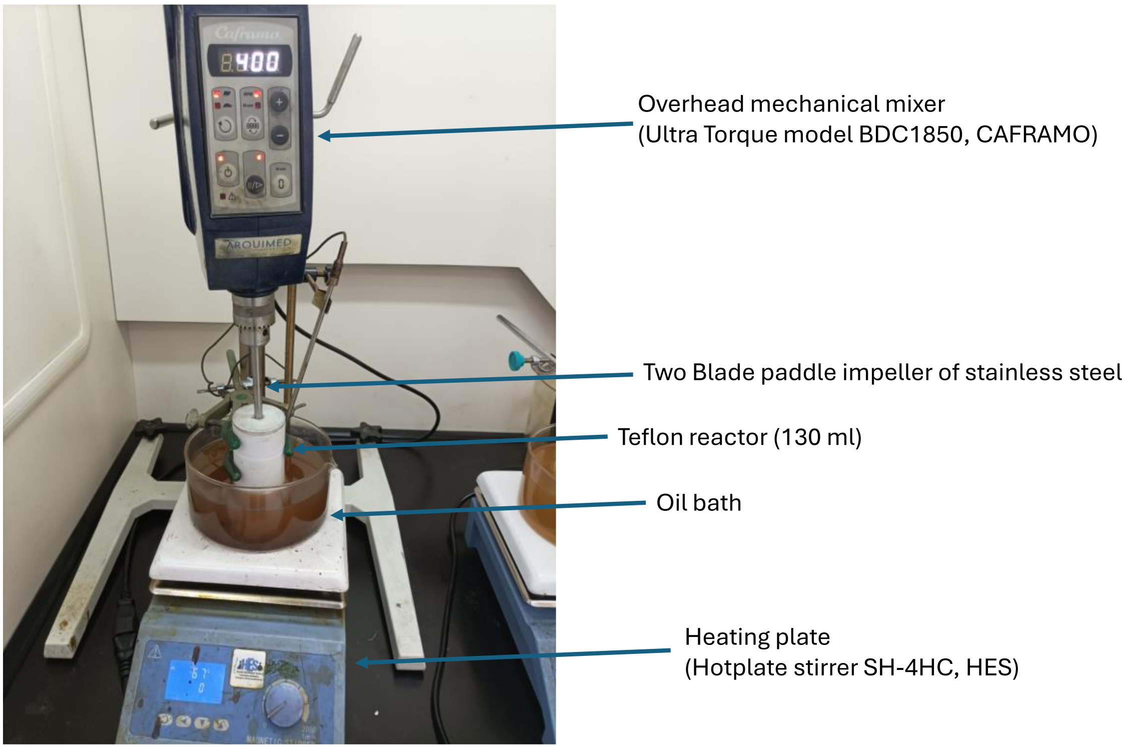

Figure S2. Teflon reactor setup for exfoliation of graphite.

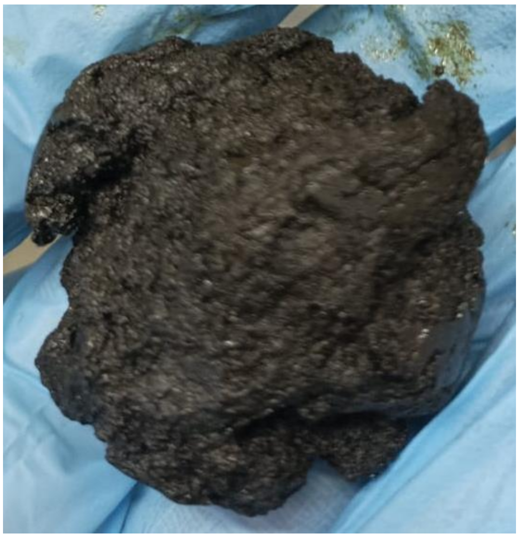

Figure S3. Insoluble PKFU-Gr Crosslinked composite after DA reaction.

Functional polyketone characterization using EA, FTIR and 1H-NMR

Table S2. Elemental analysis of PK alone and chemically modified with Am, FU, and THI at different molar ratios.

Sample	X(%)	Y (%)	CO(%)[1]
**PK**	-	100	-
**PKAm**	38	62	38
**PKFU**	60	40	60
**PKTHI**	46	54	46

[1] Di-carbonyl conversion (CO %) obtained from EA.

ATR-FTIR and ^{1}HNMR spectroscopies analysis confirmed the functionalization of PK with AM, FU and THI. Figure 1 shows the ATR–FTIR spectrum of PKAM, PKFU and PKTHI. The intensity of the carbonyl group signal (1700 cm^{-1}) decreases due to the disappearance of the 1,4-dicarbonyl moieties after the Paal–Knorr reaction. In consequence to the disappearance of this moieties the formation of the weak to moderate signals emerged between 1500 to 1600 cm^{-1} corresponding either to stretching of the C=N and C=C bonds, additionally, a stretching of the pyrrole ring is observed at 1430 cm^{-1} in all the cases [1,2]. In the PKAM, slight band in the 730 and 780 cm^{-1} are attributed to out-of-plane C-H bending of the pyrrole ring [1]. In the PKFU sample, a distinctive C-O-C signal was identified at 1070 cm^{-1}, along with furan ring deformation at 600 cm^{-1}. Additionally, C-H out-of-plane bending signals at 880, 806 and 740 cm^{-1} were identified, associated with the furan group [3,4]. Similarly, in the case of the PKTHI sample, the C-S-C signals at 1340 cm^{-1} and the thiophene ring deformation at 690 cm^{-1}, were observed. This was accompanied by C-H out-of-plane bending at 850, 830, 760 cm^{-1} indicatives of the thiophene group [1].

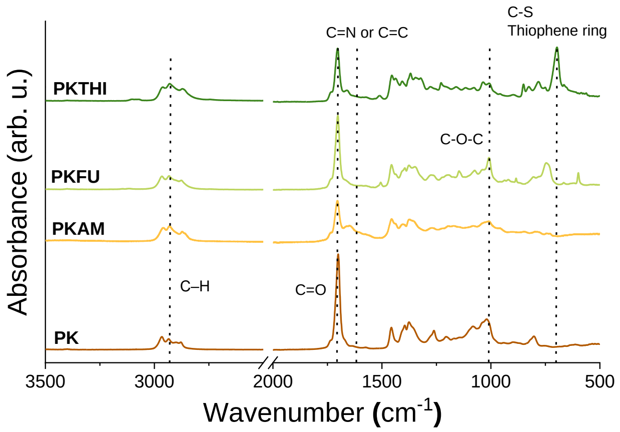

Figure S4. ATR-FTIR specta of PK, PK functionalized PKAM, PKFU, and PKTHI with the most important signals indicated.

The successful grafting of diene and aliphatic groups onto the polymer is evidenced by ^{1}H-RMN. The protons of the pyrrole ring are identified at 5.57 and 1.98 ppm [2,4,5]. In the case of PKAM, the aliphatic protons in the grafted chain are identified at 3.7, 1.3, and 0.8 ppm [1,4]. On the other

hand, the protons of PKFU corresponding to the grafted group are identified at 4.7 for the CH_2 unit connecting the pyrrole and furan ring. The signals at 7.3, 6.3, and 5.9 ppm correspond to the protons of the furan ring [3–6]. For the polymer PKTHI, the signals closely resemble those of PKFU due to the proximity of the aromatic groups and the protons belonging to the heterocycle. Specifically, the signal at 4.9 ppm is attributed to the CH_2 unit connecting the pyrrole ring and the thiophene ring. Similarly, signals are found at 7.1, 6.8, and 6.6 ppm, confirming the presence of the thiophene ring [1,5].

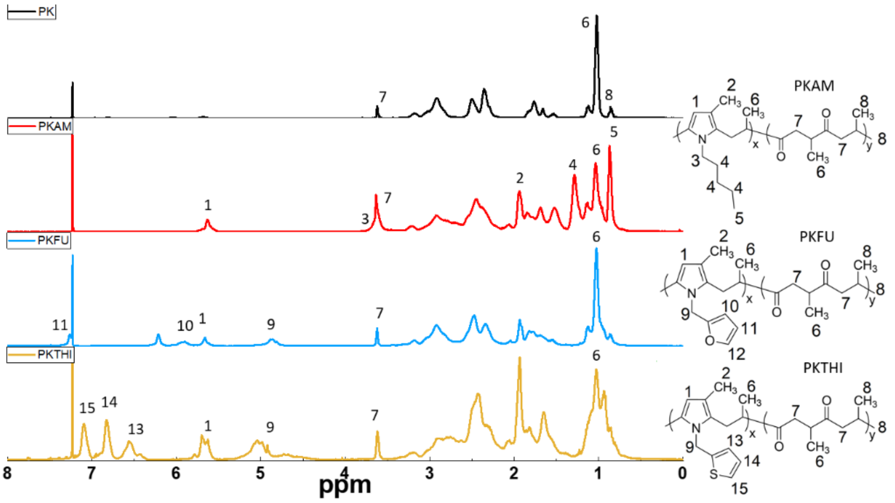

Figure S5. ^1H-NMR spectra of PK (black) and PK functionalized PKAM (red), PKFU (blue) and PKTHI (yellow), with the most important signals.

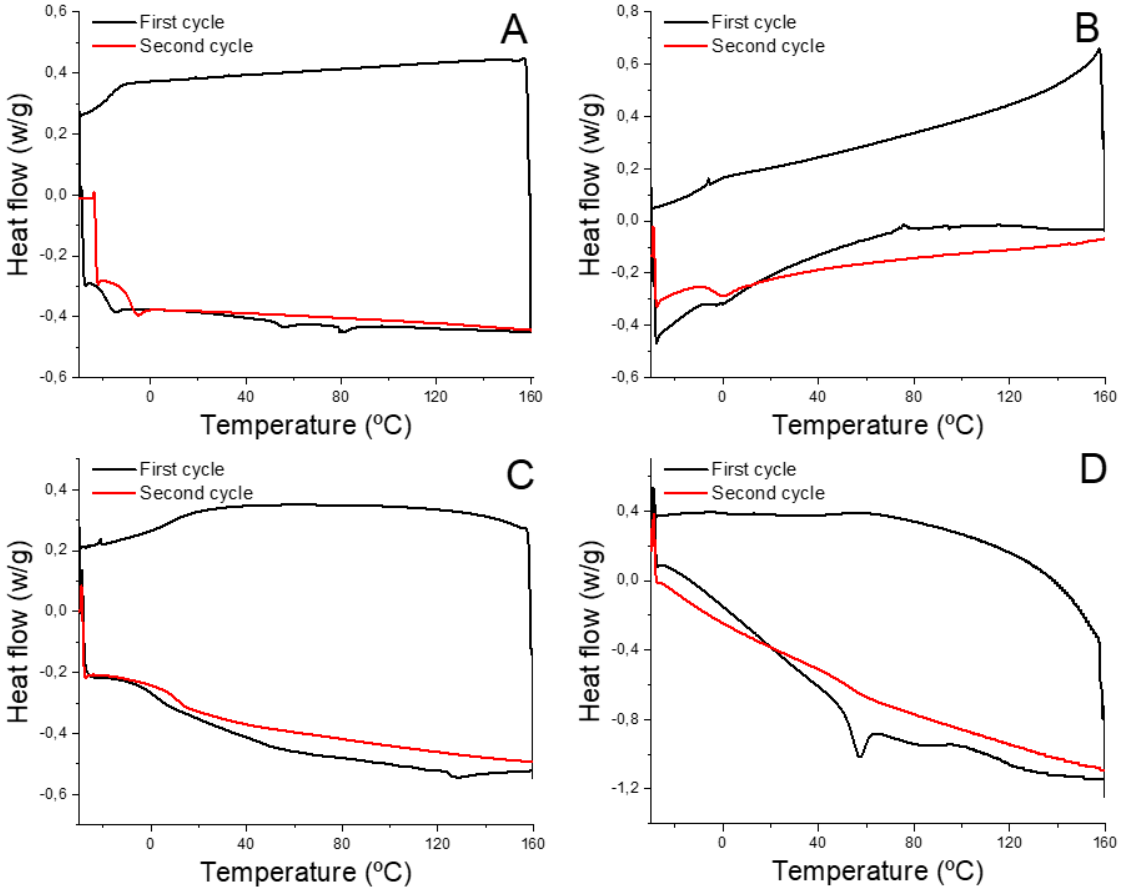

Figure S6. DSC traces of A) PK, B) PKAM, C) PKFU and D) PKTHI

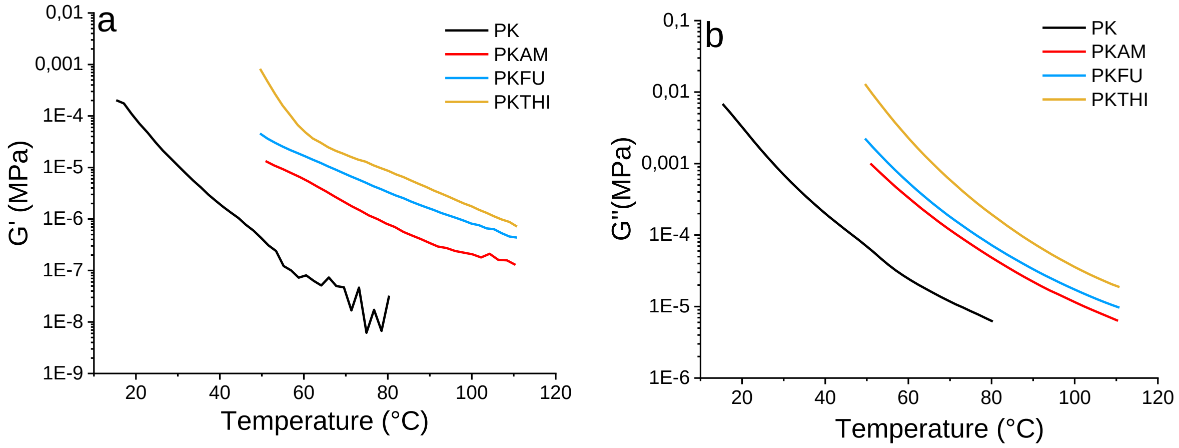

Figure S7. Rheometric test results. a) storage module and b) loss modulus of the functionalized polymers.

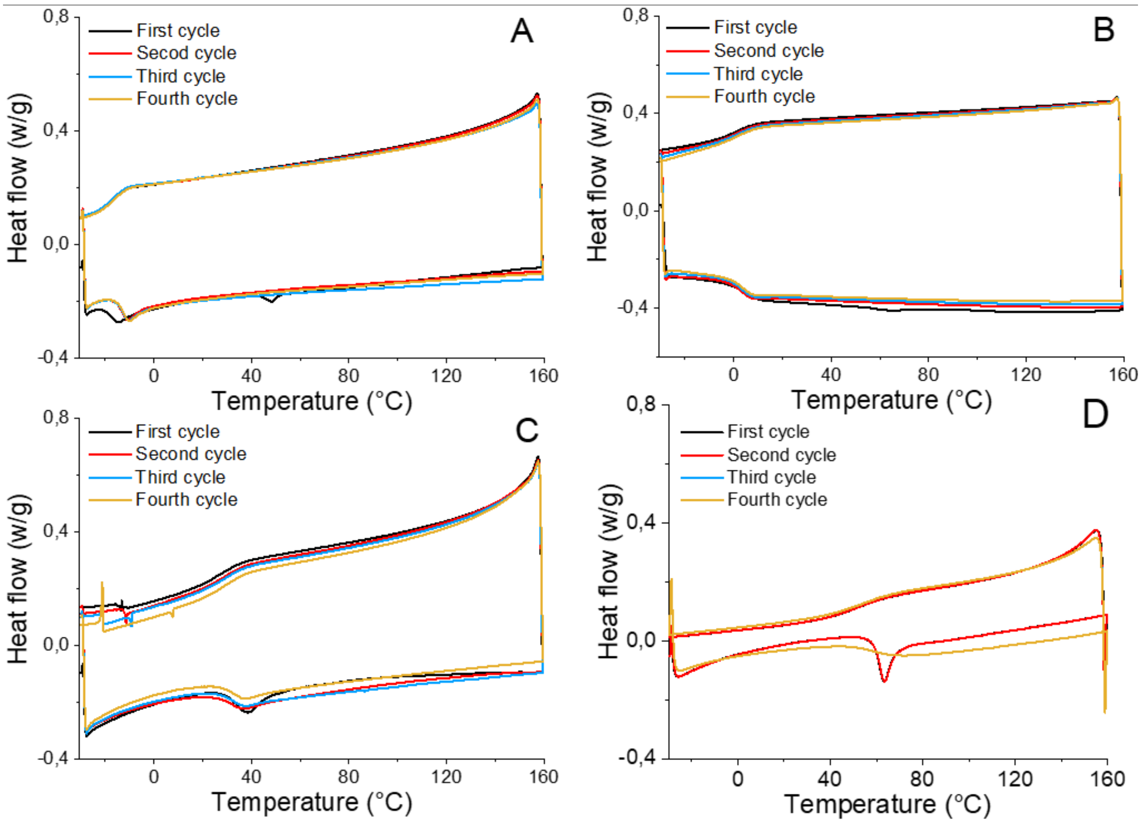

Figure S8. DSC traces of A) PKGr, B) PKAMGr, C) PKFUGr and D) PKTHIGr

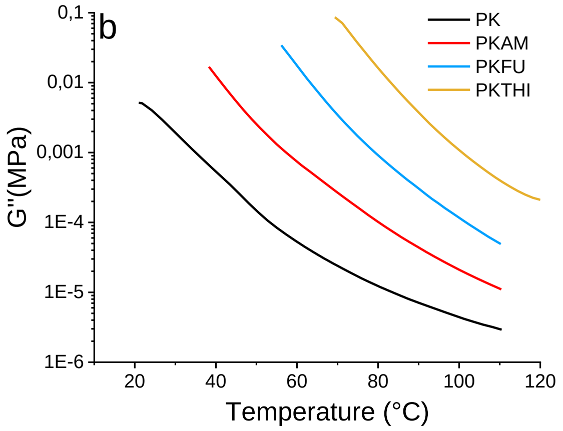

Figure S9. Rheometric test results. a) storage module and b) loss modulus of the composites.

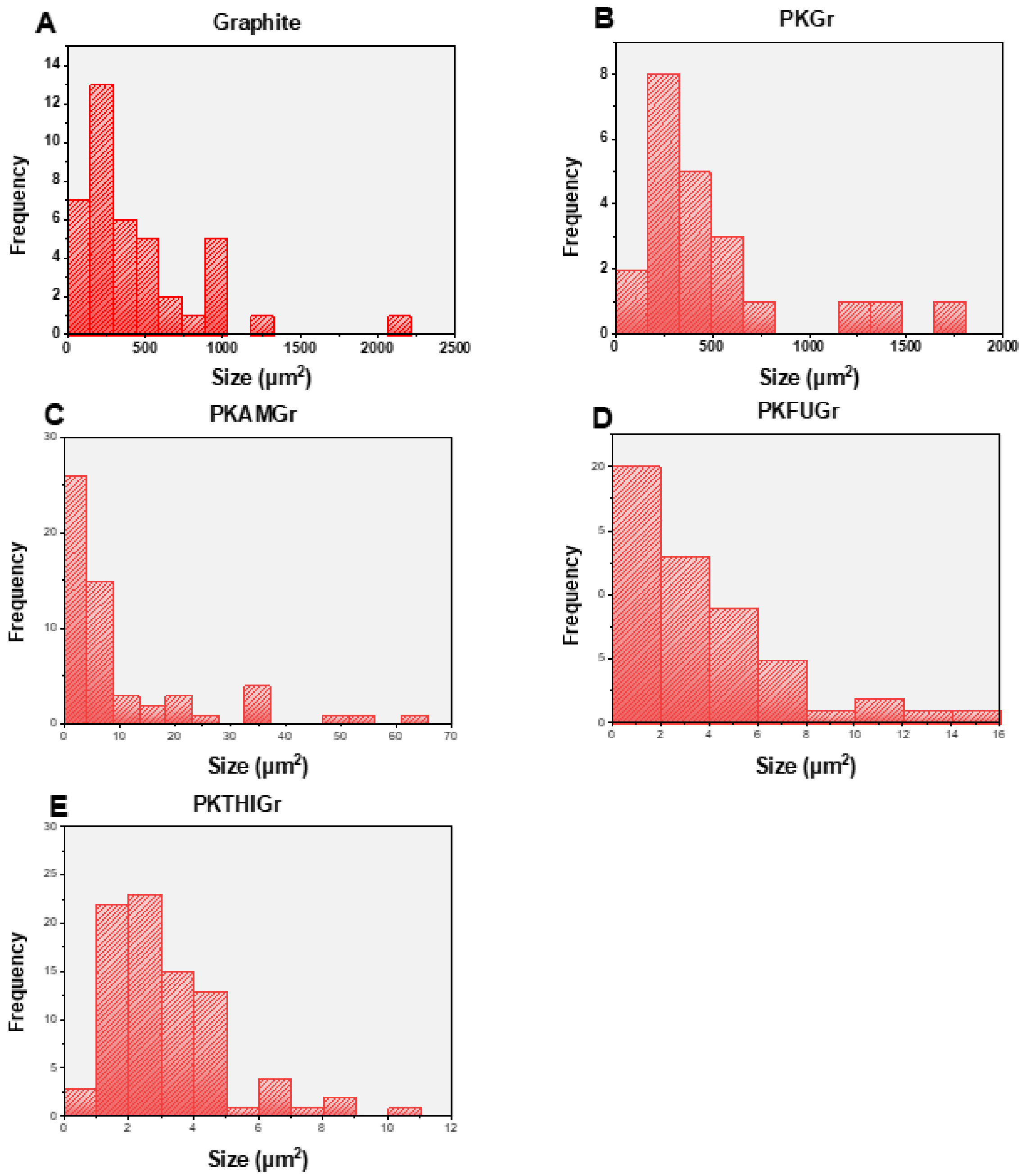

Figure S10. Size distributions obtained from SEM images. Filter of 0.45 μm: A) Graphite, B) PKGr, C) PKAMGr, D)PKFUGr, E)PKTHIGr.

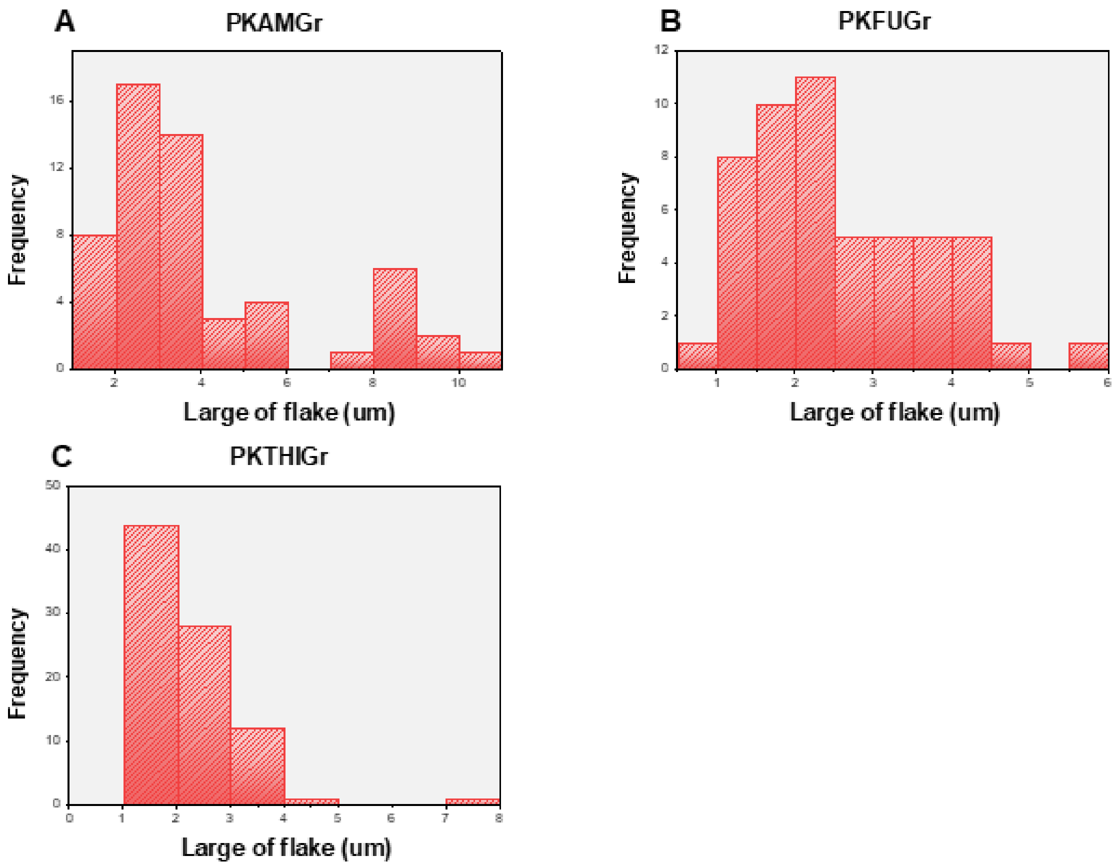

Figure S11. Size distributions obtained from SEM images. Filter of 0.45 μm, large flakes: A) PKAMGr, B)PKFUGr, C) PKTHIGr.

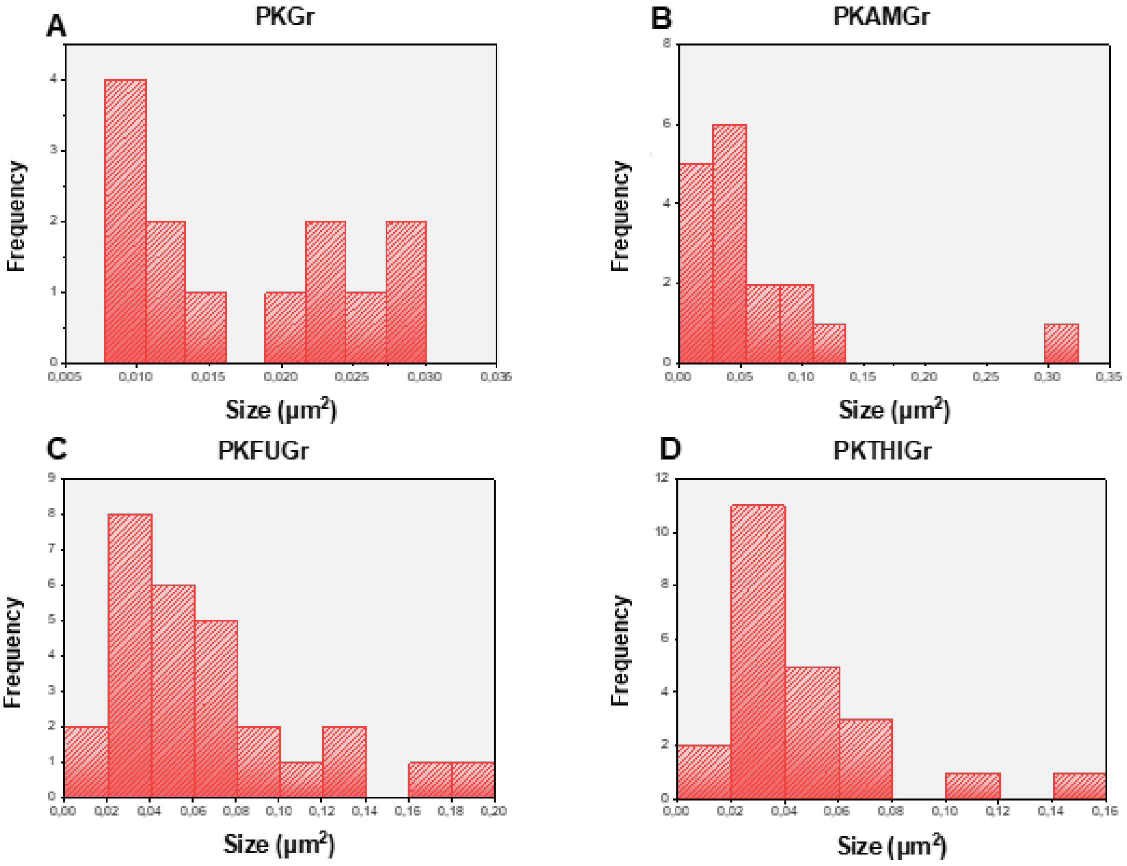

Figure S12. Size distributions obtained from SEM images. Filter 0.22 μm: A) PKGr, B) PKAMGr, C) PKFUGr, D) PKTHIGr

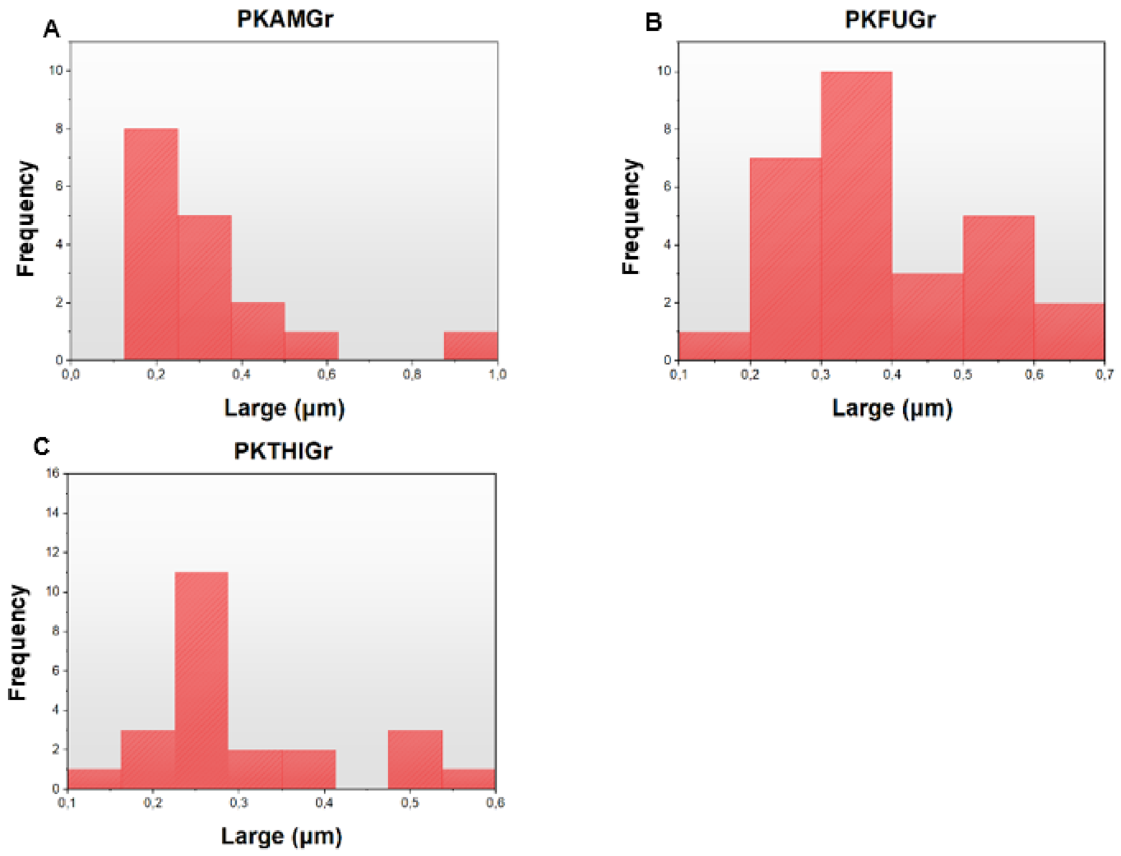

Figure S13. Size distributions obtained from SEM images. Filter 0.22 µm, large flakes: A) PKAMGr, B) PKFUGr, C) PKTHIGr.

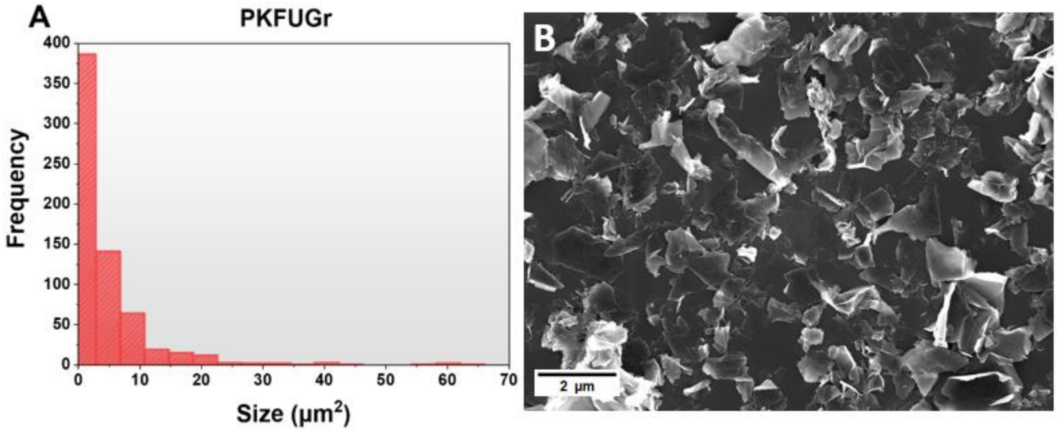

Figure S14. A) Size distribution of the exfoliated PKFUGr; B) SEM image of PKFUGr.

The histogram in the Figure S14 revealed an average particle area ranging from 0.014 µm² to 2.177 µm², representing a significant reduction—approximately 209-fold—compared to the original graphite area of 440 µm². Notably, no graphitic material with an area exceeding 70 µm² was detected, confirming the effectiveness of the exfoliation process.

Table S3. Mass yield of exfoliated graphite reached 87.9 %.

Initial mass	3.0305	Total mass recovered	2.9128
Polymer mass	2.8789	Polymer mass recovered	2.7795
graphite mass	0.1516	Graphite mass recovered	0.1333 (87.9 %)

A portion of the PK0FU 95 GR5 composite was weighed (as specified in the mass balance: 3.0305 g), theoretically containing 95 % polymer (2.8789 g) and 5 % exfoliated graphite (0.1516 g). Using this composition, the described r-DA reaction was performed. After reaction, centrifugation was employed to separate the polymer (remaining in suspension) from the exfoliated graphite, which sedimented at the bottom.

Both the polymer and the recovered graphite were dried and weighed to determine their individual masses (detailed in the mass balance). To confirm that the recovered graphite was exclusively exfoliated graphite, TGA analysis was conducted. The polymer decomposes between 300–400 °C, whereas graphite remains stable up to 700 °C. The TGA results showed a mass loss of only 1.7 %, indicating that the recovered graphite was almost entirely exfoliated.

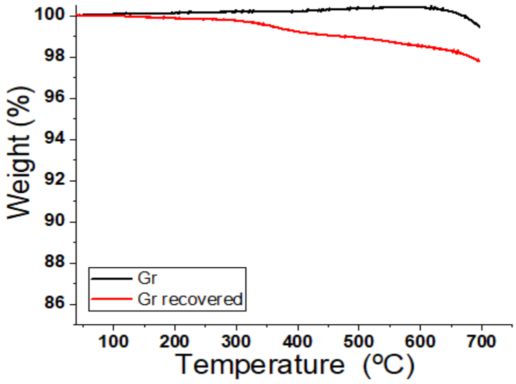

Figure S15. Thermogram obtained from untreated graphite (Gr) and graphite after exfoliation (Gr recovered).

Subsequently, SEM analysis was performed to examine the size distribution and verify the exfoliation efficiency.

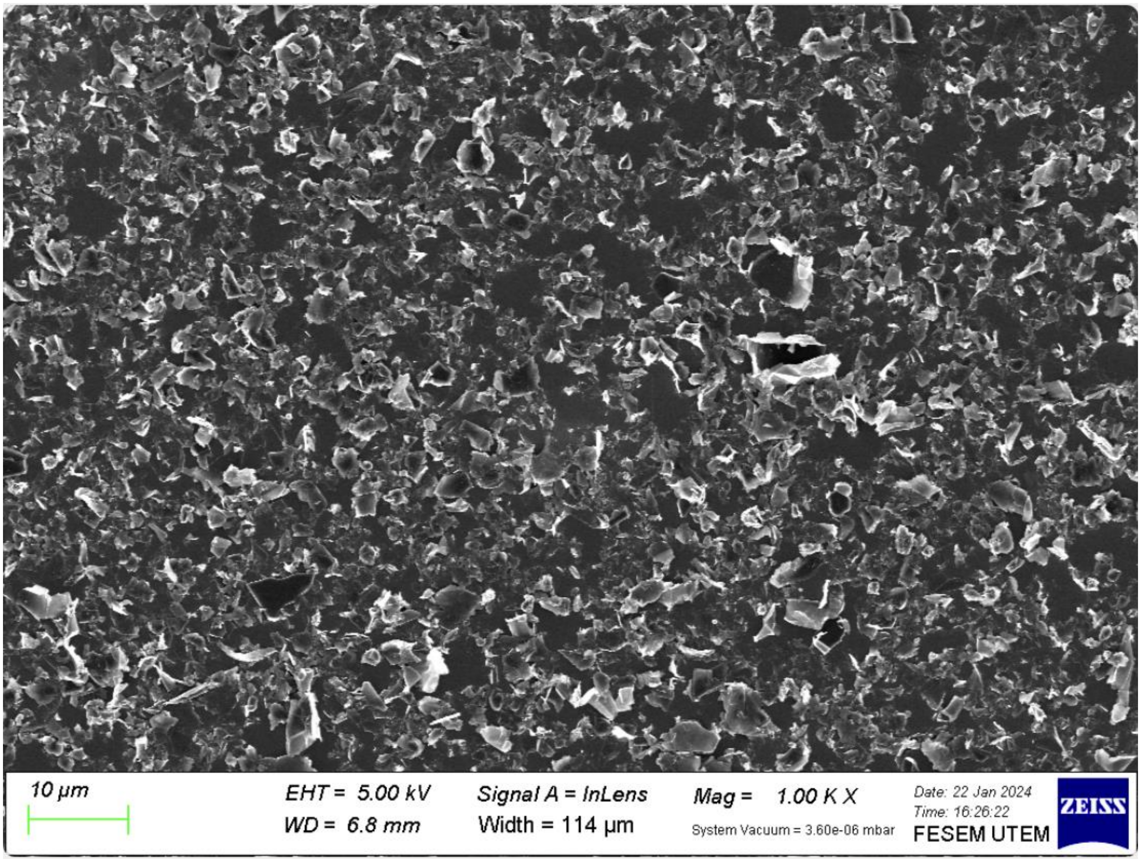

Figure S16. SEM image obtained from the recovered exfoliated graphite showing sizes less than 10 µm^2.

It is not possible to distinguish non-exfoliated graphite from exfoliated graphite based on size and morphology observed in the SEM images. While some particles appear "large", they are significantly smaller than untreated graphite chunks, which can measure up to 450 µm^2 (See figure 6A).

To analyze the exfoliated graphite, scanning transmission electron microscopy images were used. This method was selected due to its ability to reveal the exfoliation of the material more clearly, as previously observed SEM images. The samples PKFUGr and PKTHIGr were selected for analysis due to their significant results observed with the previous described techniques. Figure S16 shows nanometric sheets with multiple stacked layers of sample PKFUGr. The variability in electron density generates a grayscale in the image; the darker areas indicate a greater thickness of the exfoliated graphite material. Similarly, Figure S17 shows the PKTHIGr material, where the lamellar structures present lesser thickness, evidenced by the lighter shades of gray. These differences suggest variations in the number of stacked layers of the exfoliated graphite, and in certain areas, material folding is observed.

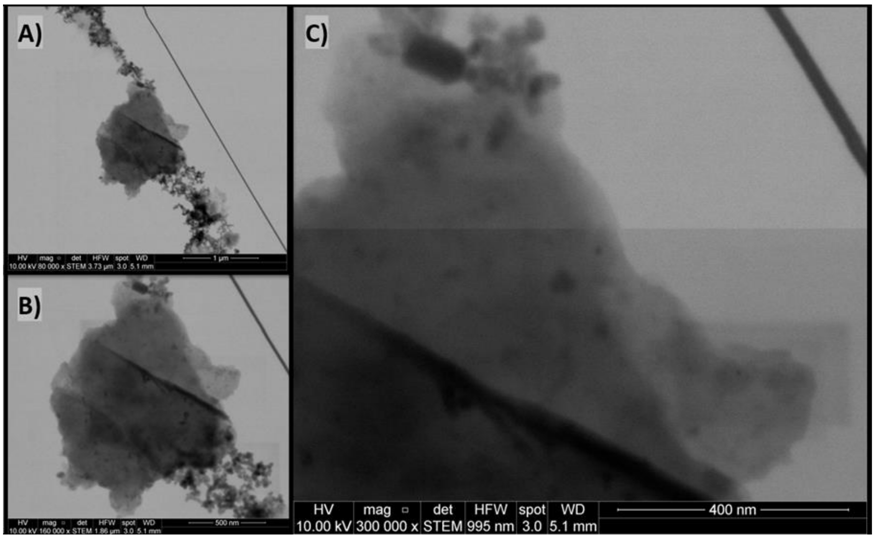

Figure S17. Images obtained from STEM analysis. PKFUGr filter system 0.45 μm

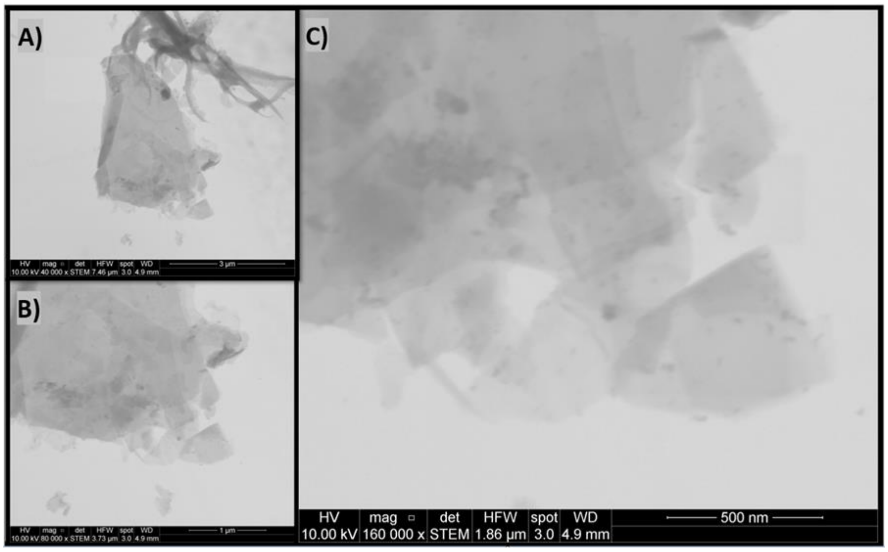

Figure S18. Images obtained from STEM analysis. PKTHIGr filter system 0.45 μm.

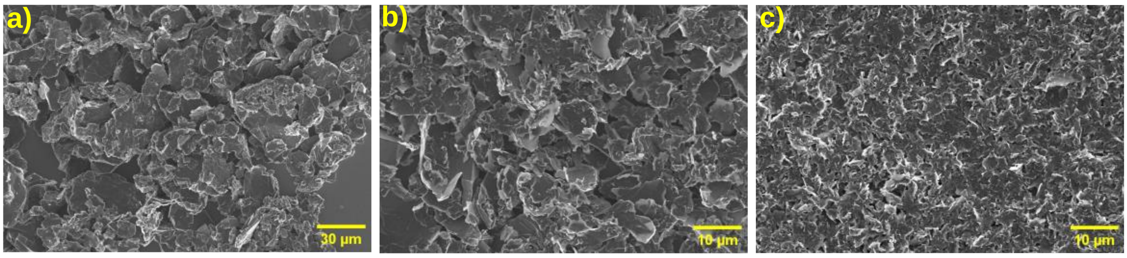

Figure S19. SEM images obtained at a magnification of a) PKGr (500x) filter 0.45 μm, b) PKAMGr (1500x) filter 0.45 μm c) PKTHIGr (1500x) filter 0.45 μm

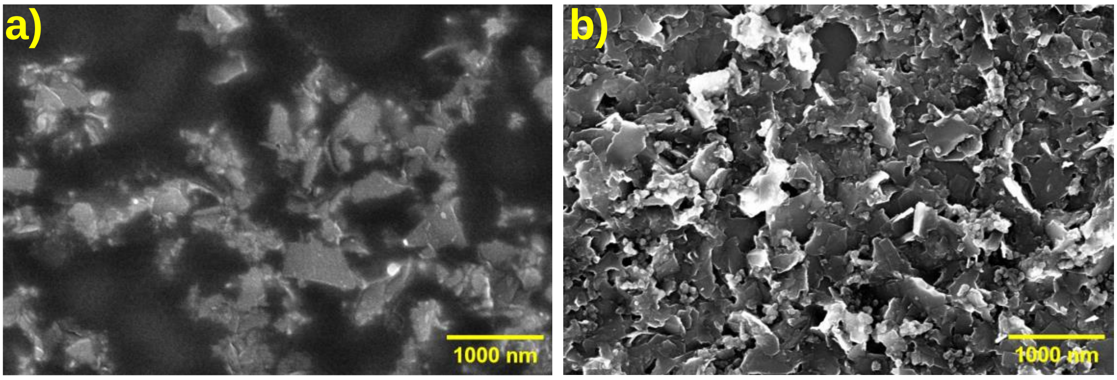

Figure S20. SEM images obtained at a magnification of 20K x a) PKAMGr b) PKTHIGr. Filter 0.22 μm.

Table S4. Mass yield thickness relative cost and scalability of exfoliated graphite and its comparison with scientific published methods.

Reference	Method	Mass Yield (%)	Thickness of Graphene/Graphite layers (nm)	Estimated Cost	Scalability
This Work	PKFU-assisted polymer exfoliation	87.9%	~30-40 nm (~80-120 layers)	Moderate (melt-processing, low solvent use)	High (easily scalable)
[7]	Liquid-phase exfoliation	~20-30%	Few nm (1-5 layers)	High (solvents, sonication)	Low (time-intensive, solvent disposal)
[8]	Electrochemical exfoliation	~60-80%	~5-20 nm	Moderate-High (electricity, electrolyte, purification)	Medium (limited by electrode area)
[9]	Mechanical milling	~40-60%	~50-100 nm	Moderate (energy consumption, equipment wear)	Medium-High (industrial milling)

References

1. Migliore, N.; Polgar, L.M.; Araya-Hermosilla, R.; Picchioni, F.; Raffa, P.; Pucci, A. Effect of the Polyketone Aromatic Pendent Groups on the Electrical Conductivity of the Derived MWCNTs-Based Nanocomposites. *Polymers (Basel)* **2018**, *8*, doi:10.3390/polym10060618.

2. Araya-Hermosilla, E.; Parlanti, P.; Gemmi, M.; Mattoli, V.; Di Pietro, S.; Iacopini, D.; Granchi, C.; Turchi, B.; Fratini, F.; Di Bussolo, V.; et al. Functionalized Aliphatic Polyketones with Germicide Activity. *RSC Adv* **2022**, *12*, 35358–35366, doi:10.1039/D2RA06396D.

3. Araya-Hermosilla, R.; Pucci, A.; Raffa, P.; Santosa, D.; Pescarmona, P.P.; Gengler, R.Y.N.; Rudolf, P.; Moreno-Villoslada, I.; Picchioni, F. Electrically-Responsive Reversible Polyketone/MWCNT Network through Diels-Alder Chemistry. *Polymers (Basel)* **2018**, *10*, doi:10.3390/polym10101076.

4. Orozco, F.; Kaveh, M.; Santosa, D.S.; Lima, G.M.R.; Gomes, D.R.; Pei, Y.; Araya-Hermosilla, R.; Moreno-Villoslada, I.; Picchioni, F.; Bose, R.K. Electroactive Self-Healing Shape Memory Polymer Composites Based on Diels–Alder Chemistry. *ACS Appl Polym Mater* **2021**, *3*, 6147–6156, doi:10.1021/acsapm.1c00999.

5. Pretsch, E.; Bühlmann, P.; Badertscher, M. Structure Determination of Organic Compounds: Tables of Spectral Data. *Structure Determination of Organic Compounds: Tables of Spectral Data* **2009**, 1–433, doi:10.1007/978-3-540-93810-1/COVER.

6. Araya-Hermosilla, R.; Fortunato, G.; Pucci, A.; Raffa, P.; Polgar, L.; Broekhuis, A.A.; Pourhossein, P.; Lima, G.M.R.; Beljaars, M.; Picchioni, F. Thermally Reversible Rubber-Toughened Thermoset Networks via Diels-Alder Chemistry. *Eur Polym J* **2016**, *74*, 229–240, doi:10.1016/j.eurpolymj.2015.11.020.

7. Paton, K.R.; Varrla, E.; Backes, C.; Smith, R.J.; Khan, U.; O'Neill, A.; Boland, C.; Lotya, M.; Istrate, O.M.; King, P.; et al. Scalable Production of Large Quantities of Defect-Free Few-Layer Graphene by Shear Exfoliation in Liquids. *Nature Materials 2014 13:6* **2014**, *13*, 624–630, doi:10.1038/nmat3944.

8. Achee, T.C.; Sun, W.; Hope, J.T.; Quitzau, S.G.; Sweeney, C.B.; Shah, S.A.; Habib, T.; Green, M.J. High-Yield Scalable Graphene Nanosheet Production from Compressed Graphite Using Electrochemical Exfoliation. *Scientific Reports 2018 8:1* **2018**, *8*, 1–8, doi:10.1038/s41598-018-32741-3.

9. León, V.; Rodriguez, A.M.; Prieto, P.; Prato, M.; Vázquez, E. Exfoliation of Graphite with Trriazine Derivatives under Ball-Milling Conditions: Preparation of Few-Layer Graphene via Selective Noncovalent Interactions. *ACS Nano* **2014**, *8*, 563–571, doi:10.1021/NN405148T.
